# Causal contextual bandits with one-shot data integration

**DOI:** 10.3389/frai.2024.1346700

**Published:** 2024-12-06

**Authors:** Chandrasekar Subramanian, Balaraman Ravindran

**Affiliations:** ^1^Robert Bosch Center for Data Science and Artificial Intelligence, Indian Institute of Technology Madras, Chennai, India; ^2^Department of Computer Science and Engineering, Indian Institute of Technology Madras, Chennai, India

**Keywords:** causality, fairness, causal contextual bandits, causal bandits, contextual bandit algorithm

## Abstract

We study a contextual bandit setting where the agent has access to causal side information, in addition to the ability to perform multiple targeted experiments corresponding to potentially different context-action pairs—simultaneously in one-shot within a budget. This new formalism provides a natural model for several real-world scenarios where parallel targeted experiments can be conducted and where some domain knowledge of causal relationships is available. We propose a new algorithm that utilizes a novel entropy-like measure that we introduce. We perform several experiments, both using purely synthetic data and using a real-world dataset. In addition, we study sensitivity of our algorithm's performance to various aspects of the problem setting. The results show that our algorithm performs better than baselines in all of the experiments. We also show that the algorithm is sound; that is, as budget increases, the learned policy eventually converges to an optimal policy. Further, we theoretically bound our algorithm's regret under additional assumptions. Finally, we provide ways to achieve two popular notions of fairness, namely counterfactual fairness and demographic parity, with our algorithm.

## 1 Introduction

Learning to make decisions that depend on context has a wide range of applications—software product experimentation, personalized medical treatments, recommendation systems, marketing campaign design, etc. Contextual bandits (Lattimore and Szepesvári, 2020) have been used to model such problems with good success (Liu et al., 2018; Sawant et al., 2018; Bouneffouf et al., 2020; Ameko et al., 2020). In the contextual bandit framework, the agent interacts with an environment to learn a near-optimal policy that maps a context space to an action space.^1^

A major challenge in wider application of contextual bandits to real world problems is the need for a large number samples, which is often prohibitively costly to obtain; indeed, this is a challenge with reinforcement learning in general (Dulac-Arnold et al., 2021). Typically, this is mitigated by considering special cases and exploiting the structures present in those settings to obtain better algorithms. Subramanian and Ravindran (2022) provided the first approach (and the only one so far) for contextual bandits where a causal graph was present as side information, in addition to the agent having the ability to perform interventions targeted on subgroups.

Specifically, in many real world scenarios, we often have some causal side information available from domain knowledge. For instance, in software product experimentation, we might know that os has a causal effect on browser, but not the other way around. Subramanian and Ravindran (2022) showed that exploiting this causal knowledge can yield better contextual bandit policies.^2^ They also introduced the notion of targeted interventions, which are interventions targeted on a specific subgroup specified by a particular assignment of values to the context variables.

This work proposes and studies a causal contextual bandits framework that has similarities with the one proposed in Subramanian and Ravindran (2022) where the agent has access to causal side information and can perform targeted interventions; the agent's objective is to minimize *simple regret* (Lattimore and Szepesvári, 2020). However, in contrast to the purely interactive setting in their work, we consider a setting where *multiple targeted interventions can be performed simultaneously in one-shot* at a cost. This, as we will see, fundamentally changes the agent's optimization problem due to the shared causal graph across the different interventions. However, importantly, it also opens up causal contextual bandits to new areas of application where it is possible to acquire additional data in one-shot at a cost and within a budget; some examples are discussed in Section 1.1. Section 1.2 provides a non-mathematical description of our framework, while Section 2.1 provides a mathematical specification of the problem. Section 1.4 provides a comparison of this work with related work.

To the best of our knowledge, there has not been any investigation on what the best way is for causal contextual bandit agents is to obtain additional experimental data in one shot and incorporate it with aim of learning a good policy.

### 1.1 Motivating examples

In software product development, product teams are frequently interested in learning the best software variant to show or roll out to each subgroup of users. To achieve this, it is often possible to conduct targeting at scale *simultaneously*, i.e., in *one shot*, for various combinations of contexts (representing user groups) and actions (representing software variants)—instead of one experiment at a time.^3^ These targeted experiments can be used to compute relevant metrics (e.g., click-through rate) for each context-action pair. Further, we might have some qualitative domain knowledge of how some context variables are causally related to others; for example, we might know that os has a causal relationship to browser; we would like to exploit this knowledge to learn better policies. This can be naturally modeled as a question of “given the causal graph, what *table* of targeted experimental data needs to be acquired and how to integrate that data, so as to learn a good policy?” here the table's rows and columns are contexts and actions, and each cell specifies the number of data samples (zero or more) required corresponding to the context-action pair. At the end of the above training phase, the agent moves to an evaluation phase (e.g., when it is deployed), where it observes contexts, and takes actions using the learned policy.

As another example, in online marketing campaign strategy, the objective is to learn the best campaign to show to each group of people.^4^ This is done by conducting experiments where different groups of people are shown different marketing campaigns simultaneously, and learning from the resultant outcomes. Further, letting context variables model the features of groups, we might have some knowledge about how some of the features are causally related to others; for example, we might know that country causally affects income. Our framework provides a natural model for this setting. Further, we might also be interested in ensuring that the campaigns meet certain criteria for fairness. For example, there might be some variables such as race that could be sensitive from a fairness perspective, and we would not want the agent to learn policies that depends on these variables. We discuss fairness implications of our algorithm in Section 5. These are just two of many scenarios where this framework provides a natural model. Two additional examples include experimental design for ads^5^ and recommendation systems.^6^

#### 1.1.1 Remark

The ideas and approach provided in this paper are not restricted to the examples mentioned above, and can be more generally applied by the wider scientific community. The scientific method, especially in the natural and social sciences, often involves performing experiments with real world entities such as people, animals or objects. Whenever there are opportunities for conducting multiple experiments in parallel (e.g., multiple people conducting social interventions at the same time on different groups) and there is some knowledge of causal relationships between the attributes of those entities (e.g., relationships between demographic attributes of beneficiaries), the framework and approach mentioned in this paper can be considered. The algorithm in this paper provides an efficient approach for designing the parallel experiments in these settings. However, the ethical aspects of such experiments have to be accounted for, before using in practice in such cases.

### 1.2 Our framework

Our framework captures the various complexities and nuances described in the examples in Section 1.1. We present an overview here; please see Section 2.1 for the mathematical formalism. The agent's interactions consist of a learning phase where the agent incurs no regret, followed by an evaluation phase where regret is measured; this is also called *simple regret* (Lattimore and Szepesvári, 2020). At the start of the learning phase, the agent is given a (possibly empty) log of offline data—consisting of context-action-reward tuples—generated from some unknown policy. The context variables are partitioned into two sets—the main set and the auxiliary set (possibly empty). The agent observes all context variables during this phase, but learns a policy that only depends on the main set of context variables; this also provides a way to ensure that the learned policy meets certain definitions of fairness (see Section 5 for a more detailed discussion). Further, the agent also has some qualitative^7^ causal side-information available, likely from domain knowledge. This causal side-information is encoded as a causal graph between contextual variables. A key implication of the causal graph is information leakage (Lattimore et al., 2016; Subramanian and Ravindran, 2022)—getting samples for one context-action pair provides information about other context-action pairs because of shared pathways in the causal graph.

Given the logged data and the causal graph, the agent's problem is to decide the set of targeted experimental samples to acquire within a budget, and then integrate the returned samples. More specifically, the agent is allowed to make a *one-shot* request for data in the form of a table specifying the number of samples it requires for each context-action pair, subject to the total cost being within the budget. The environment then returns the requested samples after conducting the targeted interventions, and the agent integrates those samples to update its internal beliefs and learned policy. After this learning phase, the agent moves to an inference or evaluation phase, where it returns an action (according to the learned policy) for every context it encounters; its regret is measured at this point. The core problem of the agent is to choose these samples in a way that straddles the trade-off between choosing more samples for context-action pairs it knows is likely more valuable (given its beliefs) and choosing more samples to explore less-seen context-action pairs—while taking into account the budget and the information leakage across all obtained samples arising from the causal graph.

### 1.3 Contributions

1. This is the first work to study how to *actively obtain and integrate* a table of *multiple samples in one-shot* in a contextual bandit setting. Further, we study this in the presence of a causal graph, making it one of the very few works to study the *utilization of causal side-information* by contextual bandit agents. See Section 1.4 for a more detailed discussion on related work, and Section 2.1 for the mathematical formalism of the problem.
2. We propose a novel algorithm (Section 2.2.3) that works by minimizing a new entropy-like measure called Υ(.) that we introduce. See Section 2.2 for a full discussion on the approach.
3. We show results of extensive experiments using purely synthetically generated data and an experiment inspired by real-world data, that demonstrate that our algorithm performs better than baselines. We also study sensitivity of the results to key aspects of the problem setting. See Section 4.
4. We also show some theoretical results. Specifically, we show that the method is sound – that is, as the budget tends to infinity, the algorithm's regret converges to 0 (Section 3.2). Further, we provide a bound on regret for a limited case (Section 3.1).
5. We discuss fairness implications of our method in Section 5. We show that it can achieve counterfactual fairness. Further, while the algorithm does not guarantee demographic parity, we provide a way to recover this notion of fairness, but with a reduction in performance.

### 1.4 Related work

Causal bandits have been studied in the last few years (e.g., Lattimore et al., 2016; Yabe et al., 2018; Lu et al., 2020), but they study this in a multi-armed bandit setting where the problem is identification of one best action. There is only one work (Subramanian and Ravindran, 2022) studying causal *contextual* bandits—where the objective is to learn a *policy* mapping contexts to actions—and this is the closest related work. While we do leverage some ideas introduced in that work in our methodology and in the design of experiments, our work differs fundamentally from this work in important ways. Subramanian and Ravindran (2022) consider a standard interactive setting where the agent can repeatedly act, observe outcomes and update its beliefs, whereas in our work the agent has a one-shot data collection option for samples from *multiple* context-action pairs. This fundamentally changes the nature of the optimization problem as we will see in Section 2.2; it also makes it a more natural model in a different set of applications, some of which were discussed in Section 1.1. Further, our work allows for arbitrary costs for collecting those samples, whereas they assume every intervention is of equal cost.

Contextual bandits in purely offline settings, where decision policies are learned from logged data, is a well-studied problem. Most of the work involves inverse propensity weighting based methods (such as Swaminathan and Joachims, 2015b,a; Joachims et al., 2018). Contextual bandits are also well-studied in purely interactive settings (see Lattimore and Szepesvári, 2020 for a discussion on various algorithms). However, in contrast to our work, none of these methods can integrate causal side information or provide a way to actively acquire and integrate new targeted experimental data.

Active learning (Settles, 2012) studies settings where an agent is allowed to query an oracle for ground truth labels for certain data points. This has been studied in supervised learning settings where the agent receives ground truth feedback; in contrast, in our case, the agent receives outcomes only for actions that were taken (“bandit feedback”). However, despite this difference, our approach can be viewed as incorporating some elements of active learning into contextual bandits by enabling the agent to acquire additional samples at a cost. There has been some work that has studied contextual bandits with costs and budget constraints (e.g., Agrawal and Goyal, 2012; Wu et al., 2015). There has also been work that has explored contextual bandit settings where the agent can not immediately integrate feedback from the environment, but can do so only in batches (Zhang et al., 2022; Ren et al., 2022; Han et al., 2020). However, all these works consider settings where the samples are obtained through a standard contextual bandit interaction—observe a context, choose an intervention, receive a reward; in contrast, our work considers *targeted* interventions where context values to determine the targeted subgroup is specified along with the intervention. Further, importantly, none of these works provide a way to integrate causal side information.

## 2 Methodology

### 2.1 Problem formalism

#### 2.1.1 Underlying model

We model the underlying environment as a causal model M, which is defined by a directed acyclic graph G over all variables (the “causal graph”) and a joint probability distribution ℙ that factorizes over G (Pearl, 2009b; Koller and Friedman, 2009). The set of variables in G consists of the action variable (*X*), the reward variable (*Y*), and the set of context variables (C). Each variable takes on a finite, known set of values; note that this is quite general, and accommodates categorical variables. C is partitioned into the set of main context variables $\left(C^{A}\right)$ and the set of (possibly empty) auxiliary context variables $\left(C^{B}\right)$. That is, $C=C^{A}\cup C^{B}$.

The agent knows only G but not M; therefore, the agent has no *a priori* knowledge of the conditional probability distributions (CPDs) of the variables.

#### 2.1.2 Protocol

In addition to knowing G, the agent also has access to logged offline data, $D_{L}=\left\{\left({\text{c}}_{i},x_{i},y_{i}\right)\right\}$, where each (**c**_*i*_, *x*_*i*_, *y*_*i*_) is sampled from M and *x*_*i*_ is chosen following some unknown policy. Unlike many prior works, such as Swaminathan and Joachims, 2015b, the agent here does *not* have access to the logging propensities.

The agent then specifies in one shot the number of samples $N_{x,{\text{c}}^{A}}$ it requires for each pair (*x*, **c**^*A*^).^8^ We denote the full table of these values by $\text{N}≜{⋃}_{x,{\text{c}}^{A}}\left\{N_{x,{\text{c}}^{A}}\right\}$. Given a (*x*, **c**^*A*^), there is an arbitrary cost $\beta \left(x,{\text{c}}^{A},N_{x,{\text{c}}^{A}}\right)$ associated with obtaining those samples. The total cost should be at most a budget *B*. For each (*x*, **c**^*A*^), the environment returns $N_{x,{\text{c}}^{A}}$ samples of the form $\left({\text{c}}^{B},y\right)\sim \mathbb{P}\left(C^{B},Y\;|\;do\left(x\right),{\text{c}}^{A}\right)$.^9^ Let's call this acquired dataset $D_{A}$. The agent utilizes $D_{A}$ along with $D_{L}$ to learn a good policy.

#### 2.1.3 Objective

The agent's objective is to learn a policy $\hat{\phi }\;:\;\text{val}\left(C^{A}\right)\to \text{val}\left(X\right)$ such that expected simple regret is minimized:


$$\begin{array}{l} Regret≜\underset{{\text{c}}^{A}}{\sum }\left[\mu_{{\text{c}}^{A}}^{*}-{\hat{\mu }}_{{\text{c}}^{A}}\right]\cdot \mathbb{P}\left({\text{c}}^{A}\right) \end{array}$$

where ϕ^*^ is an optimal policy, $\mu_{{\text{c}}^{A}}^{*}≜𝔼\left[Y|do\left(\phi^{*}\left({\text{c}}^{A}\right),{\text{c}}^{A}\right)\right]$ and ${\hat{\mu }}_{{\text{c}}^{A}}≜𝔼\left[Y|do\left(\hat{\phi }\left({\text{c}}^{A}\right),{\text{c}}^{A}\right)\right]$.

Table 1 provides a summary of the key notation used in this paper.

**Table 1:** Summary of key notation.

**Notation**	**Meaning**
*X*	Action variable
*Y*	Reward variable
$C^{A},C^{B}$	Set of main context variables and set of auxiliary context variables, respectively; so the set of all context variables is $C=C^{A}\cup C^{B}=\left\{...,C_{i},...\right\}$.
Capital letters	A random variable; e.g., *C*_1_ or *X*
Small letters	A random variable's value; e.g., *c*_1_ or *x*
Small bold font	An assignment of values to a set of random variables; for example, **c** denotes a specific choice of values taken by variables in C
$\hat{\mathbb{P}}$, $\hat{𝔼}$	Estimate of distribution ℙ and expectation 𝔼 based on current beliefs
val(V),val(V)	Set of values taken by the variable *V*, and set of variables V, respectively.
$\hat{\phi },\phi^{*}$	The learned policy and an optimal policy, respectively
Υ	Entropy-like measure used in our algorithm; defined in Equation 3
**pa**_*V*_	Value of variables in *PA*_*V*_, the parents of *V*
$N_{x,{\text{c}}^{A}}$	Number of samples requested corresponding to *X* = *x* and $C^{A}={\text{c}}^{A}$
$\beta \left(x,{\text{c}}^{A},N_{x,{\text{c}}^{A}}\right)$	Cost of acquiring $N_{x,{\text{c}}^{A}}$ samples corresponding to *X* = *x* and $C^{A}={\text{c}}^{A}$
*B*	budget
a〈B〉	If **a** is an assignment of values to A, then a〈B〉 is assignment of those values to respective variables B; a〈B〉=∅ if A∩B=∅.

#### 2.1.4 Assumptions

We assume that *X* has exactly one outgoing edge, *X* → *Y*, in G. This is suitable to express a wide range of problems such as personalized treatments or software experimentation where the problem is to learn the best action under a context, but the action or treatment does not affect context variables. We also make a commonly-made assumption (see Guo et al., 2020) that there are no unobserved confounders. Similar to Subramanian and Ravindran (2022), we make an additional assumption that simplifies the factorization in Section 2.2.2: $\left\{C\text{ confounds }C^{\prime }\in C^{A}\text{ and }Y\right\}\;\Rightarrow \;C\in C^{A}$; a *sufficient* condition for this to be true is if $C^{A}$ is ancestral.^10^ This last assumption is a simplifying assumption and can be relaxed in the future.

### 2.2 Solution approach

#### 2.2.1 Overall idea

In our approach, the agent works by maintaining beliefs^11^ regarding every conditional probability distribution (CPD) in M. It first uses $D_{L}$ to update its initial CPD beliefs; this, in itself, makes use of information leakage provided the causal graph. It next needs to choose $D_{A}$, which is the core problem. The key tradeoff facing the agent is the following: it needs to choose between allocating more samples to context-action pairs that it believes are more valuable and to context-action pairs that it knows less about. Unlike Subramanian and Ravindran (2022), it cannot interactively choose and learn, but instead has to choose the whole $D_{A}$ in one shot—necessitating the need to account for multiple overlapping information leakage pathways resulting from the multitude of samples. In addition, these samples have a cost to acquire, given by an arbitrary cost function, along with a total budget.

##### 2.2.1.1 Toward solving this

To achieve this, we define a novel function Υ(**N**) that captures a measure of overall entropy weighted by value. The idea is that minimizing Υ results in a good policy; that is, the agent's problem now becomes that of minimizing Υ subject to budget constraints. In Section 2.2.2, we formally define Υ(**N**) and provide some intuition. Later, we provide experimental support (see Section 4), along with some theoretical grounding to this intuition (see Section 3).

#### 2.2.2 The optimization problem

Determine $N_{x,{\text{c}}^{A}}$ for each (*x*, **c**^*A*^) such that


Υ(N)

is minimized, subject to


$$\underset{x,{\text{c}}^{A}}{\sum }\beta \left(x,{\text{c}}^{A},N_{x,{\text{c}}^{A}}\right)\leq B$$

where $\text{N}≜{⋃}_{x,{\text{c}}^{A}}\left\{N_{x,{\text{c}}^{A}}\right\}$. We will next define Υ(**N**).

##### 2.2.2.1 Defining the objective function Υ(**N**)

The conditional distribution ℙ(*V*|**pa**_*V*_) for any variable *V* is modeled as a categorical distribution whose parameters are sampled from a Dirichlet distribution (the belief distribution). That is, ℙ(*V*|**pa**_*V*_) = Cat(*V*; *b*_1_, ..., *b*_*r*_), where (*b*_1_, ..., *b*_*r*_)~Dir(θ_*V*|_**pa**_*V*___), and θ_*V*|_**pa**_*V*___ is a vector denoting the parameters of the Dirichlet distribution.

Actions in a contextual bandit setting can be interpreted as *do*() interventions on a causal model (Zhang and Bareinboim, 2017; Lattimore et al., 2016). Therefore, the reward *Y* when an agent chooses action *x* against context **c**^*A*^ can be thought of as being sampled according to ℙ[*Y*|*do*(*x*), **c**^*A*^]. Under the assumptions described in Section 2.1.4, we can factorize as follows:


(1) $$\begin{array}{l} 𝔼\left[Y|do\left(x\right),{\text{c}}^{A}\right]=\underset{\begin{array}{c} {\text{c}}^{B}\in \text{val}\left(C^{B}\right) \end{array}}{\sum }[\mathbb{P}\left(Y=1|x,\text{c}\langle {PA}_{Y}\rangle \right)\underset{c\in {\text{c}}^{B}}{\prod }\mathbb{P}\left(C=c|\text{c}\langle {PA}_{C}\rangle \right)] \end{array}$$

Crucially, note that our beliefs about each CPD in Equation 1 are affected by samples corresponding to multiple (*x*, **c**^*A*^) due to the shared terms in the factorization. To capture this, we construct a CPD-level uncertainty measure which we call *Q*(.):


$$\begin{array}{l} Q\left(\mathbb{P}\left[V|{\text{pa}}_{V}\right],\text{N}\right)≜\underset{x,{\text{c}}^{A}}{\sum }\left(\frac{1}{1+\ln\;\left(N_{x,{\text{c}}^{A}}+1\right)}\right) \end{array}$$

(2) $$\begin{array}{l} \text{En}{\text{t}}^{new}\left(\mathbb{P}\left[V|{\text{pa}}_{V}\right]\right){|}_{x\langle PA_{V}\rangle ={\text{pa}}_{V}\langle X\rangle ,\;{\text{c}}^{A}\langle PA_{V}\rangle ={\text{pa}}_{V}\langle C^{A}\rangle } \end{array}$$

Here Ent^*new*^ is defined in the same way as in Subramanian and Ravindran (2022), which we reproduce here. Let the length of the vector θ_*V*|_**pa**_*V*___ be *r*. Let θ_*V*|_**pa**_*V*___[*i*] denote the *i*'th entry of θ_*V*|_**pa**_*V*___. We define an object called Ent that captures a measure of our knowledge of the CPD:


$$\text{Ent}\left(\mathbb{P}\left(V|{\text{pa}}_{V}\right)\right)≜-\underset{i}{\sum }\left[\frac{\theta_{V|{\text{pa}}_{V}}\left[i\right]}{\underset{j}{\sum }\theta_{V|{\text{pa}}_{V}}\left[j\right]}\ln\;\left(\frac{\theta_{V|{\text{pa}}_{V}}\left[i\right]}{\underset{j}{\sum }\theta_{V|{\text{pa}}_{V}}\left[j\right]}\right)\right]$$

We then define


$$\text{En}{\text{t}}^{new}\left(\mathbb{P}\left(V|{\text{pa}}_{V}\right)\right)≜\frac{1}{r}\underset{i}{\sum }\text{Ent}\left(\text{Cat}\left(b_{1}^{\prime },...,b_{r}^{\prime }\right)\right)$$

where $\left(b_{1}^{\prime },...,b_{r}^{\prime }\right)\sim \text{Dir}\left(...,\theta_{V|{\text{pa}}_{V}}\left[i-1\right],\;\theta_{V|{\text{pa}}_{V}}\left[i\right]+1,\;\theta_{V|{\text{pa}}_{V}}\left[i+1\right],...\right)$.

Finally, we construct Υ(**N**) as:


(3) $$\begin{array}{l} \Upsilon \left(\text{N}\right)≜\underset{x,\text{c}}{\sum }\left[\left[\underset{V\in C^{B}\cup \left\{Y\right\}}{\sum }Q\left(\mathbb{P}\left[V|\text{c}\langle PA_{V}\rangle \right],\text{N}\right)\right]\cdot \hat{\mathbb{P}}\left(\text{c}\right)\cdot \hat{𝔼}\left[Y|x,\text{c}\langle PA_{Y}\rangle \right]\right] \end{array}$$

##### 2.2.2.2 Intuition behind *Q*(.) and Υ(.)

Intuitively, Ent^*new*^ provides a measure of entropy if *one* additional sample corresponding to (*x*, **c**^*A*^) is obtained and used to update beliefs. *Q*(.) builds on it and captures the fact that the beliefs regarding any CPD ℙ[*V*|**pa**_*V*_] can be updated using information leakage^12^ from samples corresponding to *multiple* (*x*, **c**^*A*^); it does this by selecting the relevant (*x*, **c**^*A*^) pairs making use of the causal graph G and aggregating them. In addition, *Q*(.) also captures the fact that entropy reduces non-linearly with the number of samples. Finally, Υ(**N**) provides an aggregate (weighted) resulting uncertainty from choosing $N_{x,{\text{c}}^{A}}$ samples of each (*x*, **c**^*A*^). The weighting in Υ(.) provides a way for the agent to relatively prioritize context-action pairs that are higher-value according to its beliefs.

For further intuition, note that *Q*(ℙ[*V*|**pa**_*V*_], **N**) captures the resultant uncertainty in the agent's knowledge of ℙ[*V*|**pa**_*V*_] if samples as specified by **N** are obtained and integrated into its beliefs. Υ(**N**) not only aggregates the CPD-level measure *Q*(.) to sum over all CPDs, but also adds a weighting factor $\hat{\mathbb{P}}\left(\text{c}\right)\hat{𝔼}\left[Y|x,\text{c}\langle PA_{Y}\rangle \right]$. This term ensures that the algorithm does not overallocate samples to improve knowledge of CPDs that are less “valuable” (i.e., either the context is very unlikely to be seen, or the rewards are too low).

#### 2.2.3 Algorithm

**Figure 1:**
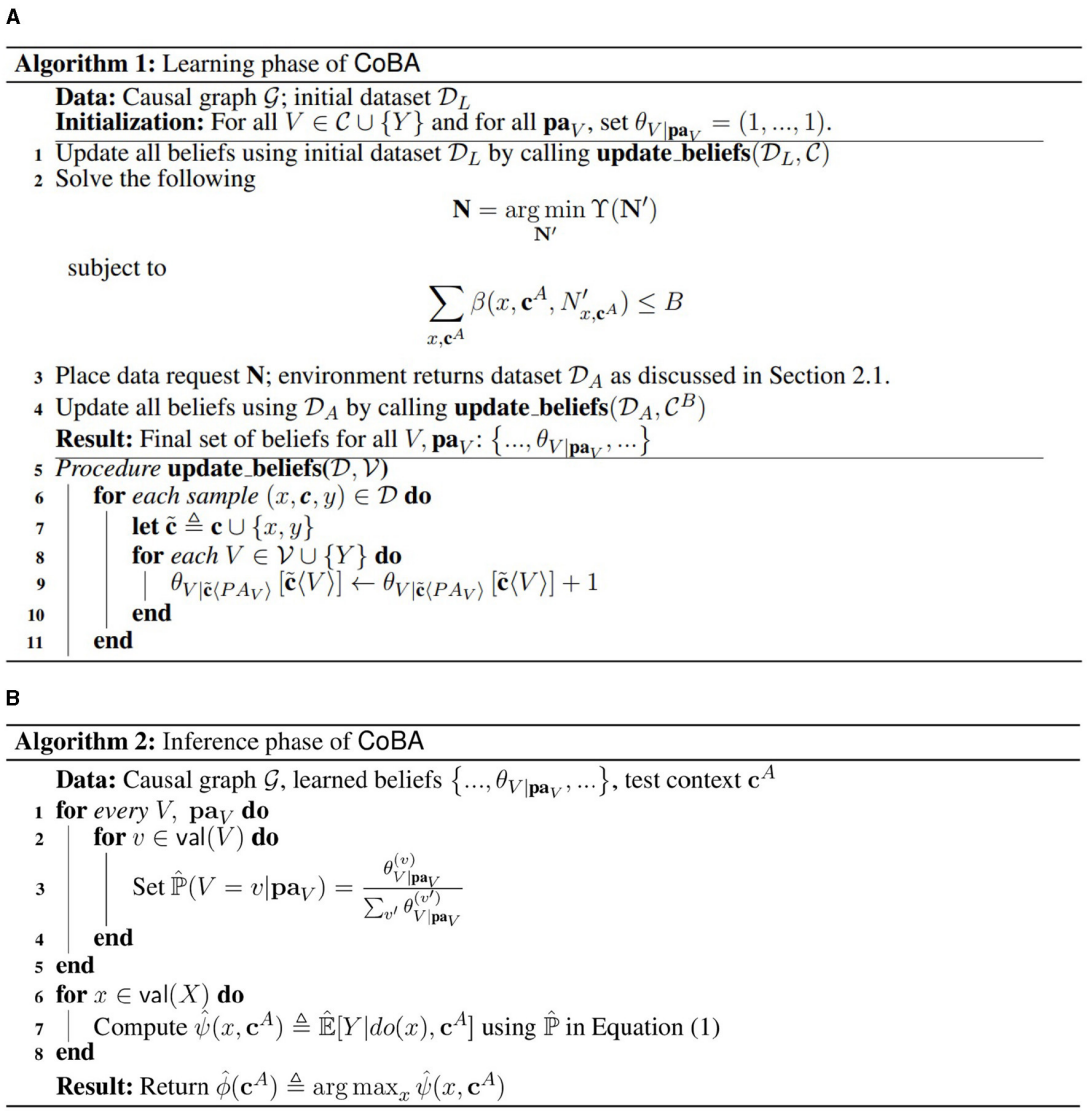
The learning **(A)** and inference **(B)** phases of our algorithm CoBA. After the learning phase is complete, the agent can be deployed to perform inference as per the inference algorithm.

The full learning algorithm, which we call CoBA, is given as Algorithm 1 (Figure 1A). After learning, the algorithm for inferencing on any test context (i.e., returning the action for the given context) is given as Algorithm 2 (Figure 1B). The core problem (Step 2 in Algorithm 1) is a nonlinear optimization problem with nonlinear constraints and integer variables. It can be solved using any of the various existing solvers; refer Section 2.2.3.1 for details on the solver used in our experiments.

##### 2.2.3.1 Solving the optimization problem in Step 2 of Algorithm 1

In our experiments in Section 4, we use the scipy.optimize.differential_evolution solver^13^ from the scipy Python library to solve the problem in Section 4.2. This solver implements differential evolution (Storn and Price, 1997), which is an evolutionary algorithm which makes very few assumptions about the problem. The scipy.optimize.differential_evolution method is quite versatile; for example, it allows the specification of the objective function as a Python callable, and also allows arbitrary nonlinear constraints of type scipy.optimize.NonlinearConstraint. However, a practitioner can use any suitable optimization algorithm or heuristic to solve this problem.

## 3 Theoretical results

We first show a regret bound for our algorithm under additional assumptions; we leave a more general bound for future work. In addition, we also show a soundness result – without any assumptions – guaranteeing convergence in the limit. Importantly, in Section 4, we perform extensive experiments where we relax the assumptions made for the regret bound and demonstrate our algorithm's empirical performance.

### 3.1 Regret bound (under additional assumptions)

In Theorem 3.1, we are interested in the case where *B* is finite. This is of interest in practical settings where the budget is usually small. We prove a regret bound under additional assumptions (A2) which we describe below. Define $m≜\underset{x,{\text{c}}^{A}}{\min}\pi \left(x|{\text{c}}^{A}\right)$, where π is the (unknown) logging policy that generated $D_{L}$; and $M_{V}≜|\text{val}\left(V\right)|$.

Theorem 3.1 (Regret bound). Under the additional assumptions (A2) mentioned below, for any 0 < δ < 1, with probability ≥ 1 − δ,


$$\begin{array}{l} Regret\in O\left(\sqrt{\left(\frac{M_{C}}{mB-\epsilon }\right)\ln\frac{M_{X}M_{C}}{\delta }}\right) \end{array}$$

where $\epsilon \in O\left(\sqrt{B\ln\left(M_{X}M_{PA_{Y}}M_{C}/\delta \right)}\right)$, ignoring terms that are constant in *B*, *m*, δ, |C| and the number of possible context-action pairs.

*Proof*. The proof closely follows the regret bound proof in Subramanian and Ravindran (2022) and adapts it to our setting. The purpose of the proof is to establish an upper bound on performance, and not to provide a tight bound. Bounding regret without these additional assumptions (A2) is left for future work.

First, we define the assumptions (A2) under which the theorem holds:

1. There is some non-empty past logged data ($|D_{L}|>0$), and it was generated by an (unknown) policy π where every action has a non-zero probability of being chosen (π(*x*|**c**^*A*^) > 0, ∀*x*, **c**^*A*^). The latter is a commonly made assumption, for example, in inverse-propensity weighting based methods.
2. $|D_{L}|\geq \alpha B$, for some constant α > 0. This is generally achievable in real world settings since we usually have fairly large logged datasets (or it is quite cheap to acquire logged data; for example, think of search logs), and for the bound to hold we technically can have a very small α as long as it is >0. We also assume that *B* is finite, as discussed earlier.
3. The cost function β is constant; without loss of generality, we let this constant be equal to 1. This is a common case in real world applications, especially when we do not have estimates of cost; in those cases, we typically assign a fixed cost to all targeted experiments.

#### 3.1.1 Expression for overall bound

First, note that Equation 3 in Subramanian and Ravindran (2022) remains the same even for our case. This is because it depends only on the factorization of 𝔼[*Y*|*do*(*x*), **c**^*A*^] (see Equation 1 in the main paper) and on the fact that in the evaluation phase the agent uses expected parameters of the CPDs (derived from its learned beliefs) to return an action for a given context.

Therefore, suppose, with probability ≥ 1 − δ_*X*,_**pa**_*Y*___,


$$\begin{array}{l} |\forall x,\hat{\mathbb{P}}\left(Y=1|X,{\text{pa}}_{Y}\right)-\mathbb{P}\left(Y=1|X,{\text{pa}}_{Y}\right)|\leq \epsilon_{X,{\text{pa}}_{Y}} \end{array}$$

and with probability ≥ 1 − δ_*C*|_**pa**_*C*___,


$$\begin{array}{l} \forall c,\;|\hat{\mathbb{P}}\left(C=c|{\text{pa}}_{C}\right)-\mathbb{P}\left(C=c|{\text{pa}}_{C}\right)|\leq \epsilon_{C|{\text{pa}}_{C}} \end{array}$$

where the expressions for δ_*X*,_**pa**_*Y*___, δ_*C*|_**pa**_*C*___, ϵ_*X*,_**pa**_*Y*___ and ϵ_*C*|_**pa**_*C*___ will be derived later in this section.

Then with probability $\geq 1-\underset{{\text{pa}}_{Y}}{\sum }\delta_{X,{\text{pa}}_{Y}}-\underset{C\in C}{\sum }\underset{{\text{pa}}_{C}}{\sum }\delta_{C|{\text{pa}}_{C}}$, for any given **c**^*A*^,


(4) $$\begin{array}{l} \text{Regret}\left({\text{c}}^{A}\right)=𝔼\left[Y|do\left(a^{*}\right),{\text{c}}^{A}\right]-𝔼\left[Y|do\left(a_{alg}\right),{\text{c}}^{A}\right]\leq 2\epsilon_{X}^{\prime }+3\underset{C\in C^{B}}{\sum }\epsilon_{C}^{\prime } \end{array}$$

where we define


$$\epsilon_{X}^{\prime }≜\underset{{\text{pa}}_{Y}}{\sum }\mathbb{P}\left({\text{pa}}_{Y}|{\text{c}}^{A}\right)\epsilon_{X,{\text{pa}}_{Y}}$$

and


$$\epsilon_{C}^{\prime }≜\underset{{\text{pa}}_{C}}{\sum }\mathbb{P}\left({\text{pa}}_{C}|{\text{c}}^{A}\right)\epsilon_{C|{\text{pa}}_{C}}$$

#### 3.1.2 Expressions for δ_*C*|_**pa**_*C*___ and ϵ_*C*|_**pa**_*C*___

Denote $M_{V}≜|\text{val}\left(V\right)|$. Let *L*_**pa**_*C*__ be the number of samples in $D_{L}$ where *PA*_*C*_ = **pa**_*C*_. Now, our starting estimate of $\hat{\mathbb{P}}\left(C=1|{\text{pa}}_{C}\right)$ using $D_{L}$ is computed as $\left(\theta_{C|{\text{pa}}_{C}}^{\left(1\right)}+1\right)/\left(L_{{\text{pa}}_{C}}+2\right)$. Since $D_{L}$ is built by observing *C*^*A*^ according to the natural distribution and choosing *X* according to some (unknown) policy, the proof of Lemma A.1 in Subramanian and Ravindran (2022) can be followed if we replace *T*′ by α*B* since $|D_{L}|\geq \alpha B$.

Therefore, suppose, with probability at least $1-\delta_{C|PA_{C}}^{L}$, it is true that


$$\begin{array}{l} \forall {\text{pa}}_{C},\;L_{{\text{pa}}_{C}}\geq \alpha B\mathbb{P}\left({\text{pa}}_{C},{\text{c}}^{A}\right)-\epsilon_{PA_{C}}^{L} \end{array}$$

If the above event is true, then it is also true that with probability at least 1 − δ_*C*|_**pa**_*C*___, it is true that


$$\begin{array}{l} \forall c,\;|\hat{\mathbb{P}}\left(c|{\text{pa}}_{C}\right)-\mathbb{P}\left(c|{\text{pa}}_{C}\right)|\leq \sqrt{\left[\frac{2}{\alpha B\mathbb{P}\left({\text{pa}}_{C},{\text{c}}^{A}\right)-\epsilon_{C|PA_{C}}^{L}}\right]\ln\left(\frac{2}{\delta_{C|{\text{pa}}_{C}}}\right)} \end{array}$$

where


$$\epsilon_{C|PA_{C}}^{L}=\sqrt{\left[\frac{\alpha B}{2}\right]\ln\left(\frac{M_{PA_{C}}}{\delta_{C|PA_{C}}^{L}}\right)},\;M_{PA_{C}}=\underset{C\in PA_{C}}{\prod }M_{C}$$

Therefore, we have that


(5) $$\begin{array}{l} \epsilon_{C|{\text{pa}}_{C}}=\sqrt{\left[\frac{2}{\alpha B\mathbb{P}\left({\text{pa}}_{C},{\text{c}}^{A}\right)-\epsilon_{C|PA_{C}}^{L}}\right]\ln\left(\frac{2}{\delta_{C|{\text{pa}}_{C}}}\right)} \end{array}$$

#### 3.1.3 Expressions for δ_*X*,_**pa**_*Y*___ and ϵ_*X*,_**pa**_*Y*___

Let *L*_*x*,_**pa**_*Y*___ be the number of samples in $D_{L}$ where (*X, PA*_*Y*_) = (*x*, **pa**_*Y*_). As before, recollect that our estimate of $\hat{\mathbb{P}}\left(Y=1|x,{\text{pa}}_{Y}\right)$ is computed as $\left(\theta_{Y|x,{\text{pa}}_{Y}}^{\left(1\right)}+1\right)/\left(L_{x,{\text{pa}}_{Y}}+2\right)$. Further, the mean of *L*_*x*,_**pa**_*Y*___ is *at least* $|D_{L}|\cdot \mathbb{P}\left({\text{pa}}_{Y},{\text{c}}^{A}\right)\cdot m>\alpha Bm\mathbb{P}\left({\text{pa}}_{Y},{\text{c}}^{A}\right)$, where $m=\underset{x,{\text{c}}^{A}}{\min}\pi \left(x|{\text{c}}^{A}\right)$ and π is the unknown logging policy. From our set of assumptions (A2), we have that π(*x*|**c**^*A*^) > 0, ∀*x*, **c**^*A*^; therefore, *m* > 0.

Given this, the proof of Lemma A.2 in Subramanian and Ravindran (2022) can be followed.

Therefore, suppose, with probability at least $1-\delta_{X,PA_{Y}}^{L}$, it is true that


$$\begin{array}{l} \forall \left(x,{\text{pa}}_{Y}\right),\;L_{x,{\text{pa}}_{Y}}\geq \alpha Bm\mathbb{P}\left({\text{pa}}_{Y},{\text{c}}^{A}\right)-\epsilon_{X,PA_{Y}}^{L} \end{array}$$

where


$$\epsilon_{X,PA_{Y}}^{L}=\sqrt{\left[\frac{\alpha B}{2}\right]\ln\left(\frac{M_{X}M_{PA_{Y}}}{\delta_{X,PA_{Y}}^{L}}\right)}$$

If the above event is true, then it is also true that with probability at least 1 − δ_*X*,_**pa**_*Y*___, it is true that


$$\begin{array}{l} \forall x,|\hat{\mathbb{P}}\left(Y=1|x,{\text{pa}}_{Y}\right)-\mathbb{P}\left(Y=1|x,{\text{pa}}_{Y}\right)|\\ \leq \sqrt{\left[\frac{2}{\alpha Bm\mathbb{P}\left({\text{pa}}_{Y},{\text{c}}^{A}\right)-\epsilon_{X,PA_{Y}}^{L}}\right]\ln\left(\frac{2M_{X}}{\delta_{X,{\text{pa}}_{Y}}}\right)} \end{array}$$

Therefore, we have that


(6) $$\begin{array}{l} \epsilon_{X,{\text{pa}}_{Y}}=\sqrt{\left[\frac{2}{\alpha Bm\mathbb{P}\left({\text{pa}}_{Y},{\text{c}}^{A}\right)-\epsilon_{X,PA_{Y}}^{L}}\right]\ln\left(\frac{2M_{X}}{\delta_{X,{\text{pa}}_{Y}}}\right)} \end{array}$$

#### 3.1.4 Final bound

Now, we can plug the Equations 5, 6 back into Equation 4, and following the same union bound trick as in Subramanian and Ravindran (2022) and some algebra, we get that for any 0 < δ < 1, with probability ≥ 1 − δ,


$$\begin{array}{l} Regret\leq 3{𝔼}_{{\text{pa}}_{Y},{\text{c}}^{A}}\left(\sqrt{\left[\frac{2}{\alpha mB\mathbb{P}\left({\text{pa}}_{Y},{\text{c}}^{A}\right)-\epsilon_{X,PA_{Y}}^{L}}\right]\ln\left(\frac{2M_{X}\left(M_{C}+|C|\right)}{\delta }\right)}\right) \end{array}$$

(7) $$\begin{array}{l} \;+3\underset{C\in C^{B}}{\sum }{𝔼}_{{\text{pa}}_{C},{\text{c}}^{A}}\left(\sqrt{\left[\frac{2}{\alpha B\mathbb{P}\left({\text{pa}}_{C},{\text{c}}^{A}\right)-\epsilon_{PA_{C}}^{L}}\right]\ln\left(\frac{2\left(M_{C}+|C|\right)}{\delta }\right)}\right) \end{array}$$

where


$$\begin{array}{l} \epsilon_{PA_{C}}^{L}=\sqrt{\left[\frac{\alpha B}{2}\right]\ln\left(\frac{M_{PA_{C}}\left(M_{C}+|C|\right)}{\delta }\right)},\\ \epsilon_{X,PA_{Y}}^{L}=\sqrt{\left[\frac{\alpha B}{2}\right]\ln\left(\frac{M_{X}M_{PA_{Y}}\left(M_{C}+|C|\right)}{\delta }\right)} \end{array}$$

It can be simplified as presented in **Theorem 3.1** as:


$$\begin{array}{l} Regret\in O\left(\sqrt{\left(\frac{M_{C}}{mB-\epsilon }\right)\ln\frac{M_{X}M_{C}}{\delta }}\right) \end{array}$$

where


$$\epsilon \in O\left(\sqrt{B\ln\frac{M_{X}M_{PA_{Y}}M_{C}}{\delta }}\right)$$

This completes the proof.

#### 3.1.5 Discussion

The bound is inversely related to $\sqrt{B}$, which is analogous to the inverse relation to $\sqrt{T}$ that other algorithms (albeit for different, but related, settings) such as Lattimore et al. (2016) and Subramanian and Ravindran (2022) have. The regret is inversely related to *m*, which can be interpreted as a measure of “hardness” of a problem (a larger *m* intuitively means the problem instance is easier); similar approaches to defining bounds in terms of hardness of the problem instance has been used in other such as Lattimore et al. (2016), Sen et al. (2017), and Yabe et al. (2018). The size of the space of possible interventions is $M_{X}M_{C}$. The bound grows with $\sqrt{M_{C}\ln\left(M_{X}M_{C}\right)}$. However, note that $m=\underset{x,{\text{c}}^{A}}{\min}\pi \left(x|{\text{c}}^{A}\right)$. This means, that even if the logging policy that generated $D_{L}$ was random (the best case), then *m* = 1/*M*_*X*_ and it forces another $\sqrt{M_{X}}$ term into the bound, thereby making the bound grow at the rate of $\sqrt{M_{X}M_{C}\ln\left(M_{X}M_{C}\right)}$; if *m* is smaller than 1/*M*_*X*_, then the bound would grow faster.

### 3.2 Soundness

Section 3.1 looked at the case where *B* is finite. In contrast, in this section, we consider the case where *B* → ∞. **Theorem 3.2** demonstrates the soundness of our approach by showing that as the budget increases, the learned policy will eventually converge to an optimal policy.

Theorem 3.2 (Soundness). As *B* → ∞, *Regret* → 0.

*Proof*. We would like to show that *Regret* → 0 as *B* → ∞. As *B* → ∞, in the limit, the problem becomes unconstrained minimization of Υ(**N**). Note that for all **N**, Υ(**N**) ≥ 0. Therefore, the smallest possible value of Υ(**N**) is 0.

First, note that $N_{x,{\text{c}}^{A}}\to \infty ,\forall \left(x,{\text{c}}^{A}\right)\;\Rightarrow \;\Upsilon \left(\text{N}\right)\to 0$. This is because ∀(*x*, **c**^*A*^),


$$N_{x,{\text{c}}^{A}}\to \infty \;\Rightarrow \;\frac{1}{1+\ln\;\left(N_{x,{\text{c}}^{A}}+1\right)}\to 0$$

which, in turn, makes *Q*(ℙ[*V*|**pa**_*V*_], **N**) → 0, ∀(*V*, **pa**_*V*_). From Equation 3, it is easy to see that this causes Υ(**N**) → 0.

Also note that $\Upsilon \left(\text{N}\right)\to 0\;\Rightarrow \;N_{x,{\text{c}}^{A}}\to \infty ,\forall \left(x,{\text{c}}^{A}\right)$. To see this, let Υ(**N**) → 0, and consider the case where there exists a (*x*, **c**^*A*^) such that $N_{x,{\text{c}}^{A}}$ is finite. That means that there is at least one term of the form


$$\frac{1}{1+\ln\;\left(N_{x^{\prime },{\text{c}}^{A\prime }}+1\right)}$$

which occurs in the *Q*(.) function of at least one CPD ℙ[*V*|**pa**_*V*_], causing *Q*(ℙ[*V*|**pa**_*V*_], **N**) > 0 since Ent^*new*^ > 0. This, in turn, causes Υ(**N**) → /0, resulting in a contradiction. Note that this makes an implicit technical assumption that ℙ[**c**] > 0, ∀**c** and that min(val(*Y*)) > 0; these are stronger assumptions than necessary, and could be weakened in the future.

Thus, $N_{x,{\text{c}}^{A}}\to \infty ,\forall \left(x,{\text{c}}^{A}\right)\Leftrightarrow \Upsilon \left(\text{N}\right)\to 0$. In other words, each (*x*, **c**^*A*^) gets a number of samples tending toward infinity if and only if Υ tends to 0. Thus, since Υ(**N**) ≥ 0, Algorithm 1 will allocate $N_{x,{\text{c}}^{A}}\to \infty ,\forall \left(x,{\text{c}}^{A}\right)$. Each CPD has at least one (*x*, **c**^*A*^) whose samples will be used to update its beliefs in Algorithm 1 (due to the technical assumption mentioned above). This means that each CPD will have its beliefs updated a number of times approaching infinity. Thus, for any (*V*, **pa**_*V*_), $\hat{\mathbb{P}}\left[V|{\text{pa}}_{V}\right]\to \mathbb{P}\left[V|{\text{pa}}_{V}\right]$. As a result, we have that $\hat{\mathbb{P}}\to \mathbb{P}$.

Since the agent's policy constructed using ℙ will necessarily be optimal, we have that *Regret* → 0. This completes the proof.

## 4 Experimental results

### 4.1 Baselines and experimental setup

#### 4.1.1 Baselines

There are no existing algorithms that directly map to our setting. Therefore, we construct a set of natural baselines and study the performance of our algorithm CoBA against them. These baselines cover standard strategies for allocation without a way to capture information leakage explicitly (which our algorithm exploits). EqualAlloc allocates an equal number of samples to all (*x*, **c**^*A*^); this provides a good distribution of samples to all context-action pairs. MaxSum maximizes the *total* number of samples summed over all (*x*, **c**^*A*^). PropToValue allocates a number of samples to (*x*, **c**^*A*^) that is proportional to $\hat{\mathbb{P}}\left({\text{c}}^{A}\right)\cdot \hat{𝔼}\left[Y|do\left(x\right),{\text{c}}^{A}\right]$; this allocates relatively more samples to context-action pairs that are more “valuable” based on the agent's current beliefs. All baselines first involve updating the agent's starting beliefs regarding the CPDs of M using $D_{L}$ (same as Step 1 of Algorithm 1) before allocating samples for active obtainment as detailed above. After $D_{A}$ is returned by the environment, all baselines use it update their beliefs (same as Step 4 of Algorithm 1).

#### 4.1.2 Experiments

Similar to Subramanian and Ravindran (2022), we consider a causal model M whose causal graph G consists of the following edges: *C*_1_ → *C*_0_, *C*_0_ → *X, C*_0_ → *Y, X* → *Y*. We let $C^{A}=\left\{C_{1}\right\}$ and $C^{B}=\left\{C_{0}\right\}$.

**Figure 2:**
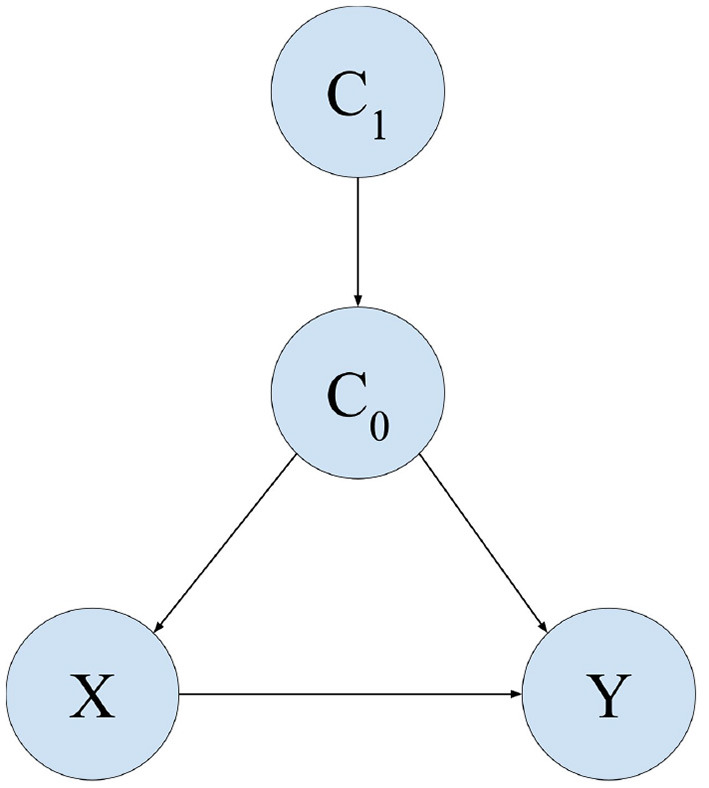
Causal graph used in all experiments except Experiment 3.

The causal graph is illustrated in Figure 2. We use this causal graph for all experiments except Experiment 3.

Experiments 1 and 2 analyze the performance of our algorithm in a variety of settings, similar to those used in Subramanian and Ravindran (2022). Experiment 3 analyzes the performance of the algorithm on a setting calibrated using real-world CRM sales-data provided in Subramanian and Ravindran (2022). For details of all the parameterizations, please refer to the Supplementary material. Experiments 4 through 7 analyze sensitivity of our algorithm's performance to various aspects of the problem setting. In all experiments, except Experiment 6, we set the cost function β(.) to be proportional to the number of samples – a natural definition of cost; in Experiment 6, we analyze sensitivity to cost function choice. Section 4.6 reports results of Experiment 1 and 2 for larger values of *B* (until all algorithms converge), providing empirical evidence of our algorithm's improved *asymptotic behavior*. Further experiments providing more insights into *why* our algorithm performs better than baselines are discussed in Section 4.7. A few additional experiments for intuition are provided in the Supplementary material.

#### 4.1.3 Remark

If the specific parameterization of M were given *a priori*, it is possible to come up with an algorithm that performs optimally in that particular setting. However, the objective is to design a method that performs well *overall* without this *a priori* information. Consider the relative performance of the baselines in Experiments 2 and 3. We will see that while EqualAlloc performs better than MaxSum and PropToValue in Experiments 3 (Section 4.4), it performs worse than those two in Experiment 2 (Section 4.3). However, our algorithm performs better than all three baselines in all experiments, corroborating our algorithm's overall better performance.

### 4.2 Experiment 1 (representative settings)

Different parameterizations of M can produce a wide range of possible settings. Given this, the first experiment studies the performance of our algorithm over a set of “*representative settings*.” Each of these settings has a natural interpretation; for example, $C^{A}$ could represent the set of person-level features that we are learning a personalized treatment for, or it could represent the set of customer attributes over which we're learning a marketing policy. The settings capture the intuition that high-value contexts (contexts for which, if the optimal action is learned, high expected rewards accrue to the agent) occur relatively less frequently (say, 20% of the time), but that there can be variation in other aspects. Specifically, the variations come from the number of different values of **c**^*A*^ over which the 20% probability mass is spread, and in how “risky” a particular context is (e.g., difference in rewards between the best and worst actions). For details of the parameterizations, please refer to the Supplementary material. The number of samples in the initial dataset $D_{L}$ is kept at $0.5\cdot |\text{val}\left(C^{A}\right)|\cdot |\text{val}\left(X\right)|$. We consider a uniformly exploring logging policy for $D_{L}$; that is, context variables for each sample are realized as per the natural distribution induced by M, but *X* is chosen randomly. In each run, the agent is presented with a randomly selected setting from the representative set. Results are averaged over 50 independent runs; error bars display ±2 standard errors.

**Figure 3:**
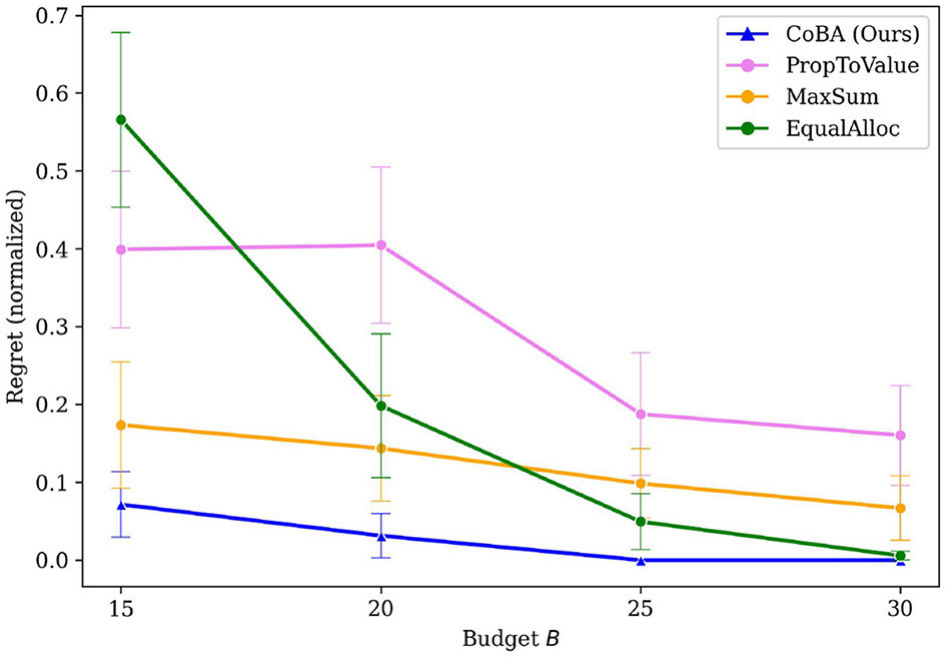
Mean regrets normalized to [0, 1] for Experiment 1 (Section 4.2). Our algorithm performs better than all baselines, with gap in performance being higher for smaller budgets.

Figure 3 provides the results of this Experiment. It plots the value of regret (normalized to [0, 1] since different settings have different ranges for regret) as budget *B* increases. We see that our algorithm performs better than all baselines. Our algorithm also retains its relatively lower regret at all values of *B*, providing empirical evidence of overall better regret performance.

### 4.3 Experiment 2 (randomized parameters)

To ensure that the results are not biased due to our choice of the representative set in Experiment 1, this experiment studies the performance of our algorithm when we *directly randomize the parameters* of the CPDs in each run, subject to realistic constraints. Specifically, in each run, we (1) randomly pick an *i* ∈ {1, ..., ⌊|val(*C*_1_)|/2⌋}, (2) distribute 20% of the probability mass randomly over the smallest *i* values of *C*_1_, and (3) distribute the remaining 80% of the mass over the remaining values of *C*_1_. The smallest *i* values of *C*_1_ have higher value (i.e., the agent obtains higher rewards when the optimal action is chosen) than the other *C*_1_ values. Intuitively, this captures the commonly observed 80–20 pattern (for example, 20% of the customers often contribute to around 80% of the revenue); but we randomize the other aspects. For details of all the parameterizations, please refer to the Supplementary material. Averaging over runs provides an estimate of the performance of the algorithms on expectation. The number of samples in the initial dataset $D_{L}$ is kept at $0.25\cdot |\text{val}\left(C^{A}\right)|\cdot |\text{val}\left(X\right)|$. The results are averaged over 50 independent runs; error bars display ±2 standard errors.

**Figure 4:**
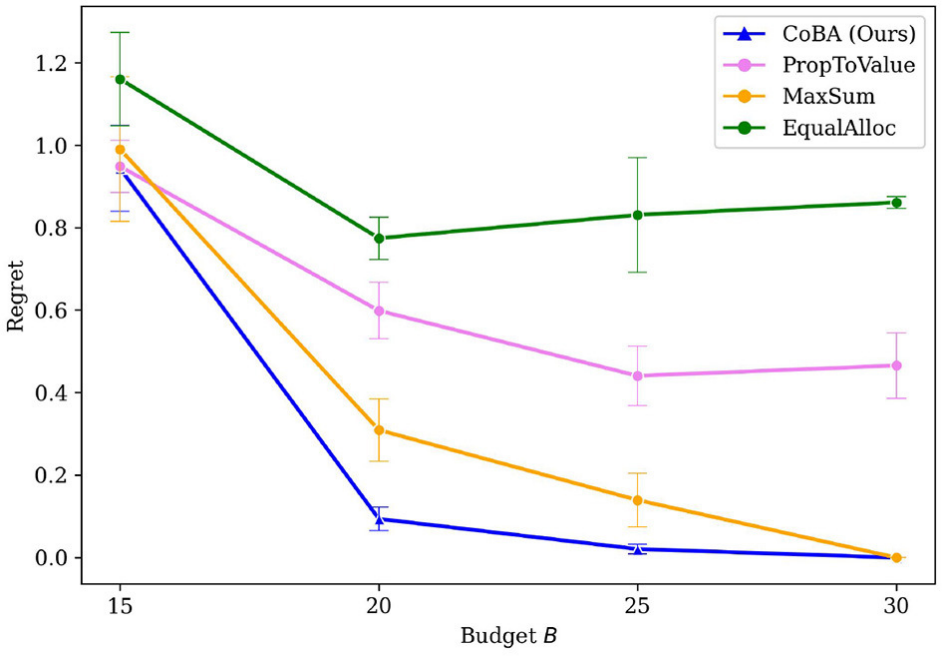
Mean regrets for Experiment 2 (Section 4.3). Our algorithm performs better than all baselines.

Figure 4 shows that our algorithm performs better than all baselines in this experiment. Our algorithm also demonstrates overall better regret performance by achieving the lowest regret for every choice of *B*.

### 4.4 Experiment 3 (calibrated using real-world data)

While Experiments 1 and 2 study purely synthetic settings, this experiment seeks to study the performance of our algorithm in *realistic scenarios*. We use the same causal graph used in the real world-inspired experiment in Section 4.2 of Subramanian and Ravindran (2022) and calibrate the CPDs using the data provided there. For parameterizations, refer to the Supplementary material.

**Figure 5:**
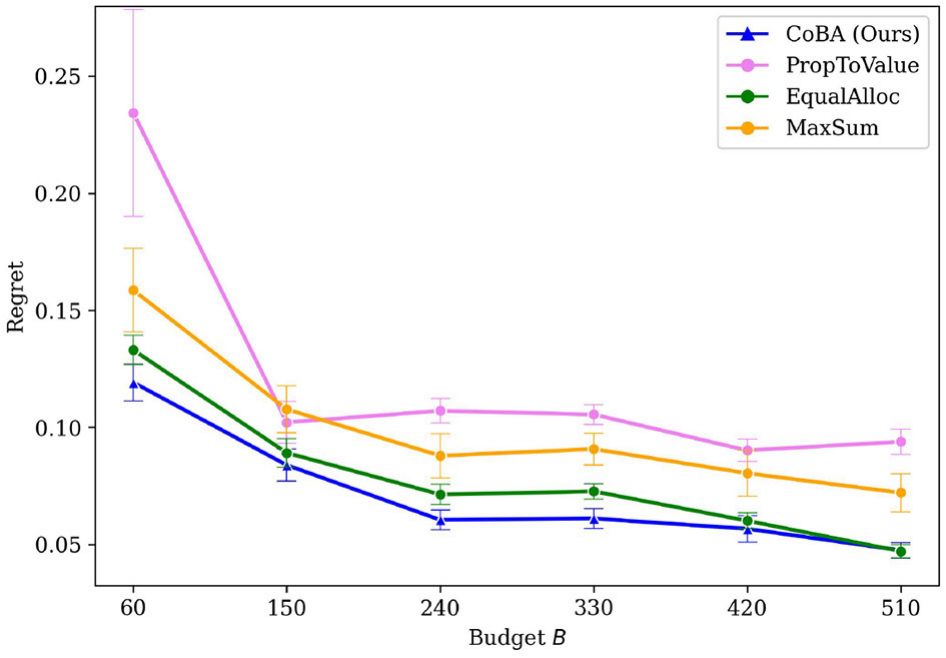
Results of the real-world data inspired experiment (Experiment 3, Section 4.4). Our algorithm achieves better mean regrets than baselines.

The objective is to learn a policy that can assist salespeople by learning to decide how many outgoing calls to make in an ongoing deal, given just the type of deal and size of customer, so as to maximize a reward metric. The variables are related to each other causally as per the causal graph [Figure 3a in Subramanian and Ravindran (2022)]. The number of samples in the initial dataset $D_{L}$ is kept at $0.125\cdot |\text{val}\left(C^{A}\right)|\cdot |\text{val}\left(X\right)|$. The results are averaged over 50 independent runs; error bars display ±2 standard errors. Figure 5 shows the results of the experiment. Our algorithm performs better than all other algorithms in this real-world inspired setting as well. Further, it retains its better performance at every value of *B*.

### 4.5 Experiments 4 through 7 (robustness to problem setting)

Experiments 4 through 7 study *sensitivity of the results to key aspects* that define our settings. To aid this analysis, instead of regret, we consider a more aggregate measure which we call AUC. For any run, AUC is computed for a given algorithm by summing over *B* the regrets for that algorithm; this provides an approximation of the area under the curve (hence the name). We then study the sensitivity of AUC to various aspects of the setting or environment.

#### 4.5.1 Experiment 4 (“narrowness” of M)

We use the term “narrowness” informally. Since our algorithm CoBA exploits the information leakage in the causal graph, we expect it to achieve better performance when there is more leakage. To see this, suppose we do a forward sampling (Koller and Friedman, 2009) of M; then, intuitively, more leakage occurs when more samples require sampling overlapping CPDs. For this experiment, we proxy this by varying |val(*C*_0_)| while keeping |val(*C*_1_)| fixed. The rest of the setting is the same as in Experiment 2. A lower |val(*C*_0_)| means that the causal model is more “squeezed” and there is likely more information leakage. The results are averaged over 50 independent runs; error bars display ±2 standard errors.

**Figure 6:**
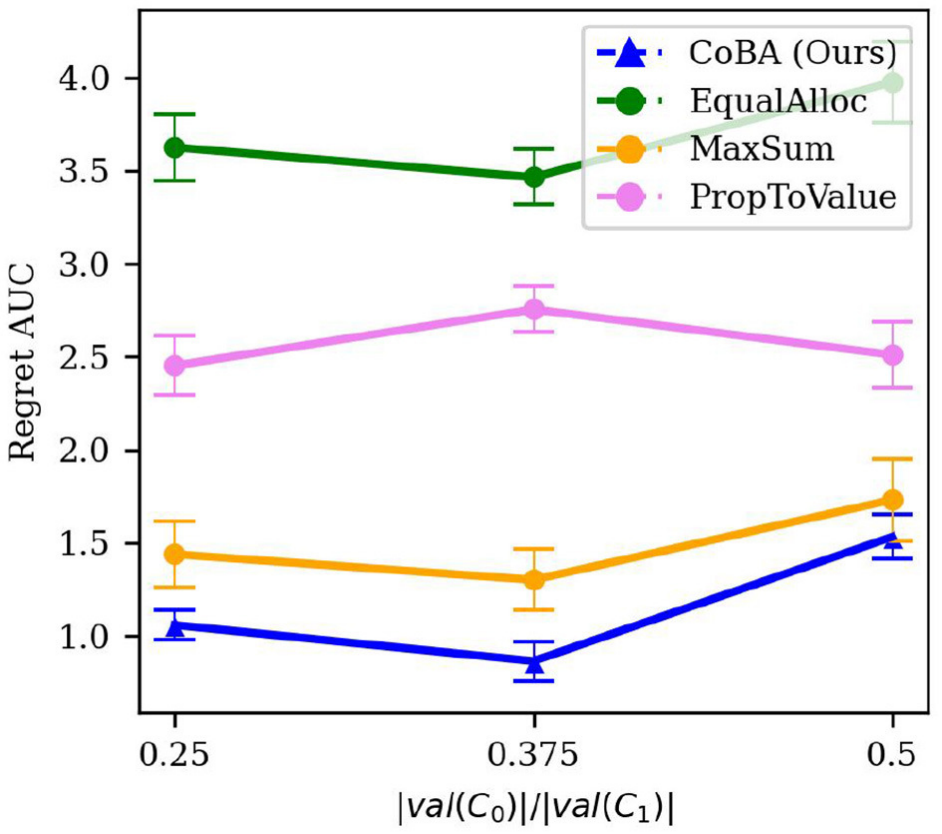
Results of the experiment studying robustness to narrowness of M (Experiment 4, Section 4.5.1). It shows the relation between |val(*C*_0_)|/|val(*C*_1_)| and Regret AUC. Our algorithm's performance remains similar (within each other's the confidence interval) for lower values of |val(*C*_0_)|/|val(*C*_1_)|, but significantly worsens when |val(*C*_0_)|/|val(*C*_1_)| = 0.5. However, our algorithm continues to perform better than all baselines for all values of |val(*C*_0_)|/|val(*C*_1_)|.

Figure 6 shows the results of this experiment. We see that our algorithm's performance remains similar (within each other's the confidence interval) for |val(*C*_0_)|/|val(*C*_1_)| ∈ {0.25, 0.375}, but significantly worsens when |val(*C*_0_)|/|val(*C*_1_)| = 0.5. However, our algorithm continues to perform better than all baselines for all values of |val(*C*_0_)|/|val(*C*_1_)|.

#### 4.5.2 Experiment 5 (size of initial dataset)

The number of samples in the initial dataset $D_{L}$ would impact the algorithm's resulting policy, for any given *B*. Specifically, we would expect that as the cardinality of $D_{L}$ increases, regret reduces. For this experiment, we consider a uniformly exploring logging policy, and vary $\left|D_{L}\right|$ by setting it to be $k\cdot |\text{val}\left(C^{A}\right)|\cdot |\text{val}\left(X\right)|$, where *k* ∈ {0, 0.25, 0.5}. The rest of the setting is the same as in Experiment 2. The results are averaged over 50 independent runs; error bars display ±2 standard errors.

**Figure 7:**
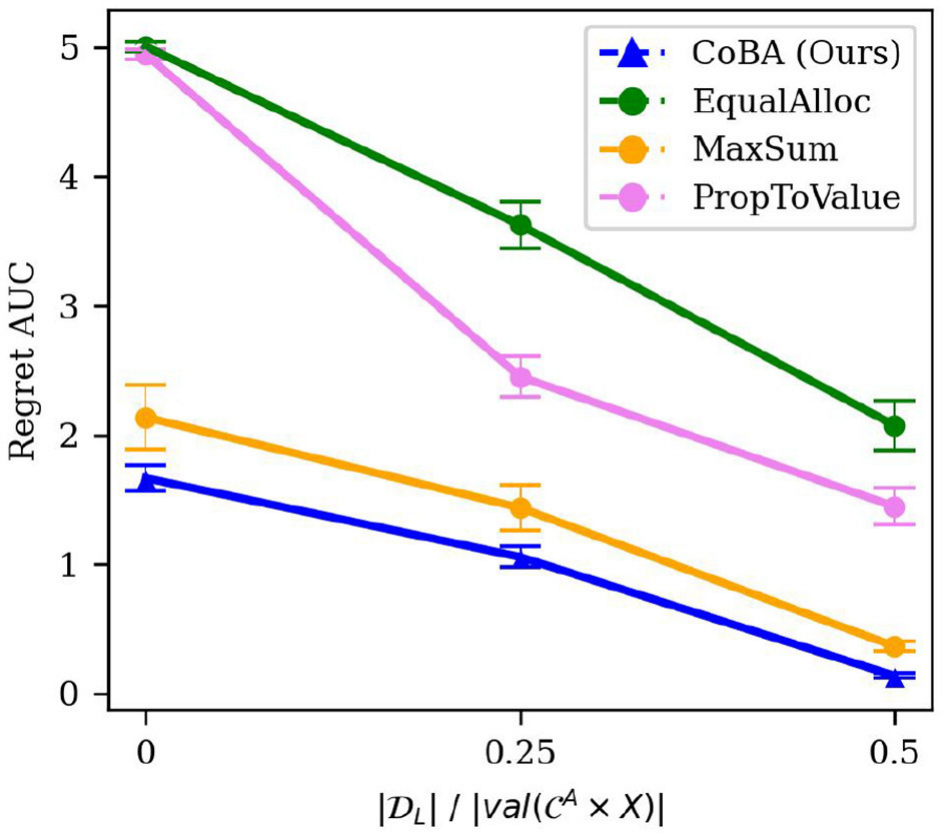
Results of the experiment studying robustness to size of the initial dataset (Experiment 5, Section 4.5.2). It shows the relation between $\left|D_{L}|/|\text{val}\left(C^{A}\times X\right)\right|$ and Regret AUC. As expected, the performance of all algorithms improve with increase in $\left|D_{L}\right|$. Importantly, our algorithm performs better than all baselines in all these settings.

The results are shown in Figure 7. We would expect the performance of all algorithms improve with increase in *k* since that would give the agent better starting beliefs; this, indeed, is what we observe. Importantly, our algorithm performs better than all baselines in all these settings. Figure 7 broken down by *B* is provided in the Supplementary material.

#### 4.5.3 Experiment 6 (choice of β)

Though we allow the cost function to be arbitrary, this experiment studies our algorithm's performance under two natural choices of β(.) to test its robustness: (1) a constant cost function; that is, $\beta \left(x,{\text{c}}^{A},N_{x,{\text{c}}^{A}}\right)\propto N_{x,{\text{c}}^{A}}$, and (2) cost function that is inversely proportional to the likelihood of observing the context naturally (i.e., rarer samples are costlier); that is, $\beta \left(x,{\text{c}}^{A},N_{x,{\text{c}}^{A}}\right)\propto \frac{N_{x,{\text{c}}^{A}}}{\mathbb{P}\left[{\text{c}}^{A}\right]}$. The rest of the setting is the same as in Experiment 2. The results are averaged over 50 independent runs; error bars display ±2 standard errors.

**Figure 8:**
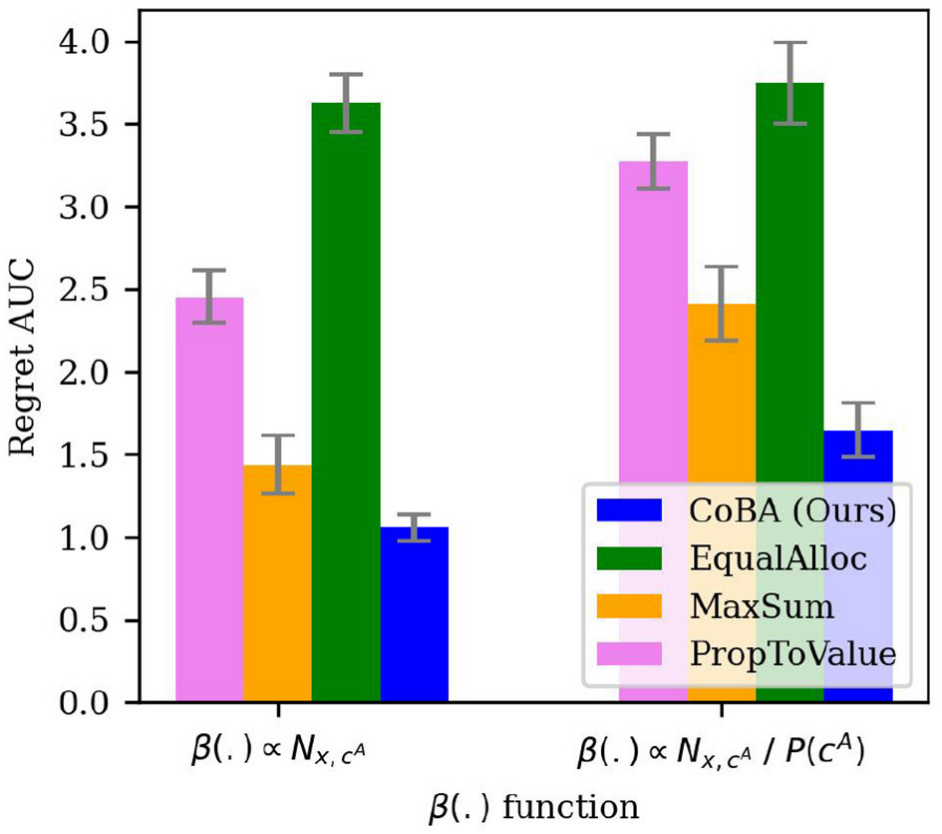
Results of the experiment studying robustness of Regret AUC of our algorithm to the choice of β (Experiment 6, Section 4.5.3). Though, as expected, the choice of cost function affects performance of all algorithms, our algorithm performs better than all algorithms for both cost function choices.

Figure 8 shows the results. As expected, the choice of cost function does affect performance of all algorithms. However, our algorithm performs better than all algorithms for both cost function choices.

#### 4.5.4 Experiment 7 (misspecification of G)

In real-world applications, the true underlying causal graph may not always be known. In this experiment, we study the impact of mis-specification of G on the performance of our algorithm. Note that the formalism described in Section 2.1 does not necessitate that the agent knows the true underlying causal graph, but rather only that it knows a causal graph such that ℙ factorizes according to it. This means that the graph G that the agent knows might include additional arrows not present in the true underlying graph. Intuitively, using such an imperfect graph would result in worsened performance by our algorithm since there are less overlapping information pathways to exploit.

**Figure 9:**
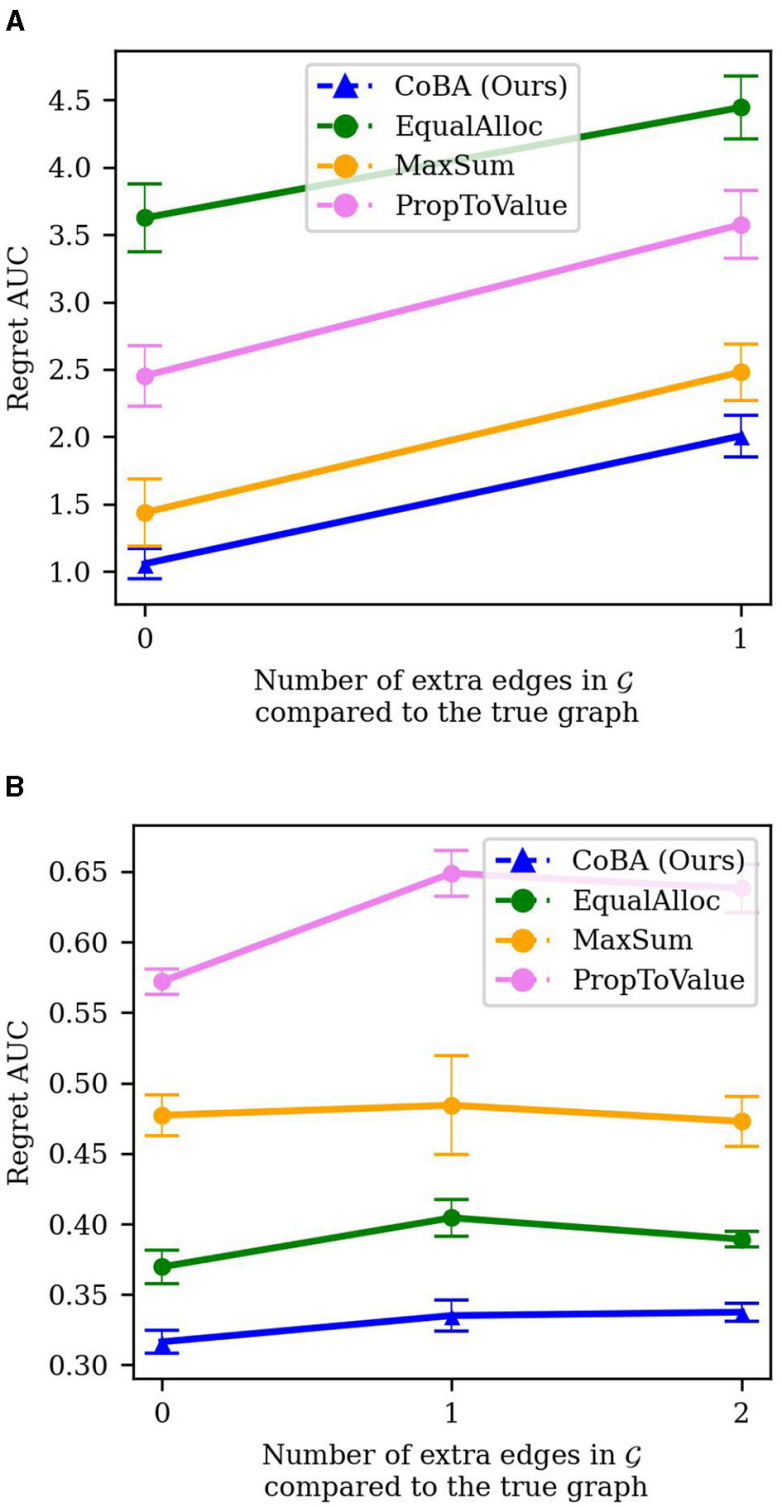
**(A)** Experiment 7a results (misspecification of G in Experiment 2). Though, as expected, imperfect knowledge of the true underlying graph results in reduced performance of our algorithm, it retains its relative better performance. **(B)** Experiment 7b results (misspecification of G in Experiment 3). All algorithms experience mild degradation in performance; our algorithm retains its relative performance compared to baselines.

In Experiment 7a, we study this effect empirically by comparing results of Experiment 2 to the same experiment but with the causal graph having an extra edge: *C*_1_ → *Y*. The results are averaged over 25 independent runs; error bars display ±2 standard errors. Figure 9A shows the results of the experiment. As expected, performance of our algorithm degrades when there is imperfect knowledge of the true underlying graph. However, our algorithm continues to perform better than all baselines, while also maintaining a similar difference in regret AUC compared to the baselines. Figure 9A broken down by *B* is provided in the Supplementary material.

In Experiment 7b, we perform a similar analysis to the real-world inspired experiment presented in Section 4.4. Specifically, we compare the case where the true causal graph is known with the cases where there is a misspecification of the causal relationships between the context variables. To capture this, we add one edge (*C*_1_ → *C*_0_) to G and then one more edge (*C*_2_ → *C*_1_); we compare the performance under these two settings to the case where the true causal graph is known. The results are averaged over 25 independent runs; error bars display ±2 standard errors. The results in Figure 9B shows the results. In this case, the deterioration in performance due to misspecification of G is quite small for all algorithms. However, our algorithm continues to perform better than all baselines; it performs the best when the true graph is known.

### 4.6 Regret behavior for large values of *B*

Figures 3, 4 provided regret behavior for small values of *B*. We are primarily interested in such small-budget behavior since that occurs more commonly in practice; for example, budgets exclusively for experimentation in software teams in often quite low.

**Figure 10:**
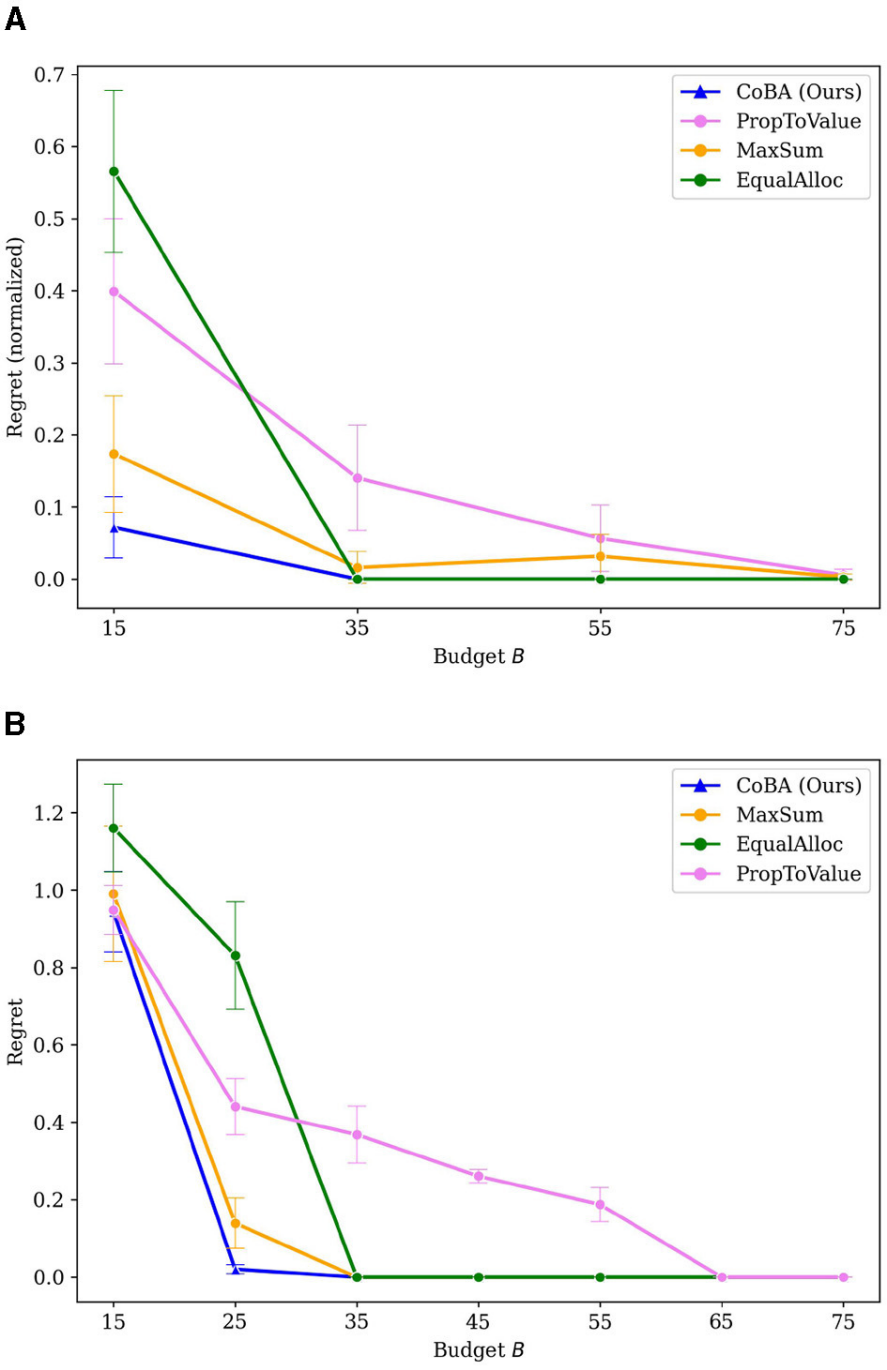
**(A)** Results of Experiment 1 (provided in Section 4.2) extended for large values of *B*. **(B)** Results of Experiment 2 (provided in Section 4.3) extended for large values of *B*.

However, it is also interesting to look at regret behavior as *B* becomes large. Specifically, we increase *B* large enough that all algorithms converge to optimal (or very close to optimal). We do this for Experiments 1 and 2. Figures 10A, B provide the results. Note that the Figures 3, 4 just zoom into these plots for small *B* (i.e., *B* between 15 and 30).

PropToValue is the slowest to converge to the optimal policy in both instances, though it demonstrates better low-budget behavior than EqualAlloc. MaxSum has the best low budget behavior among the baselines because it maximizes the total number of samples within that low budget; it, as *B* gets larger, EqualAlloc catches up (and even outperforms it) as it explores the context-action space better. In both experiments, however, our algorithm converges to an optimal policy faster than all baselines.

### 4.7 Intuition for better performance of our algorithm

As discussed in Section 2.2, our algorithm balances the trade off between allocating more samples to context-action pairs that are higher value according to its beliefs and allocating more samples for exploration, while taking into account information leakage due to the causal graph. To understand this in more detail, we consider Setting 1 of Experiment 1, and zoom into the case where *B* = 20. We do 50 independent runs and plot the frequency of choosing samples containing different value of *C*_1_. We show this for our algorithm and all baselines (except EqualAlloc since it is obvious how it allocates).

**Figure 11:**
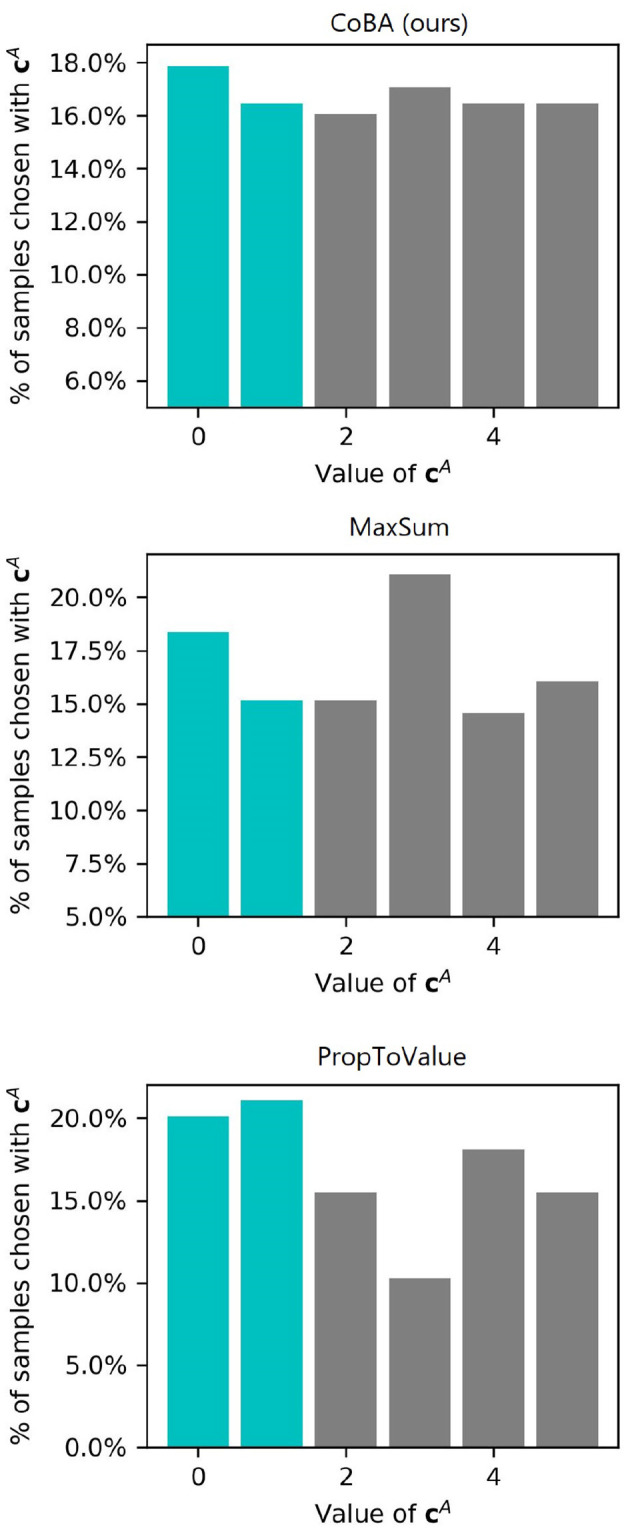
Frequency of choosing or encountering each value of $C^{A}$. Highlighted in teal color are the “high-value” contexts (i.e., contexts for which learning the right actions provides higher expected rewards).

Figure 11 shows the results of this experiment. MaxSum allocates lesser number of samples than our algorithm to the two context values (*C*_1_ ∈ {0, 1}) that are high value. PropToValue over-allocates to these two context values, resulting in poor exploration of other contexts. Our algorithm, in contrast, allocate relatively more to the high-value contexts, while also maintaining good exploration of other contexts.

## 5 Fairness results

Fairness is becoming an increasingly important angle to discuss when designing machine learning algorithms. A common way to approach fairness is to ensure some subset of variables (assumed given to the algorithm), called “sensitive variables,” is not discriminated against. Specific formal definitions of this discrimination give rise to different notions of fairness in literature (Grgić-Hlača et al., 2016; Dwork et al., 2012; Kusner et al., 2017; Zuo et al., 2022; Castelnovo et al., 2022).

### 5.1 Counterfactual fairness

Counterfactual fairness is a commonly used notion of individual fairness. Intuitively, a *counterfactually fair* mapping from contexts to actions ensures that the actions mapped to an individual (given by a specific choice of values for the context variables) are the same in a counterfactual world where a subset W⊆C of sensitive contexts is changed.

For example, if a loan decision policy is counterfactually fair with respect to gender, then a a male applicant would have had the same probability of being granted the loan *had he been* a different gender. Counterfactual fairness requires the knowledge of the causal graph to be able to establish, making it difficult to use in practice; however, when causal graphs are available, it is a powerful notion of individual-level fairness.

In our case, counterfactual fairness can be achieved by setting $C^{B}$ to contain all the sensitive attributes; that is, by letting $C^{B}⊇W$.

#### 5.1.1 Proof of counterfactual fairness

To prove counterfactual fairness, first note that the learned policy is a map $\hat{\phi }\;:\;\text{val}\left(C^{A}\right)\to \text{val}\left(X\right)$; during inference, for any given **c**^*A*^, the value of *X* is intervened to be set to $\hat{\phi }\left({\text{c}}^{A}\right)$. Following the notation in Pearl (2009a), we let ${\hat{\phi }}_{C^{B}\leftarrow {\text{c}}^{B\prime }}\left({\text{c}}^{A}\right)$ denote $\hat{\phi }\left({\text{c}}^{A}\right)$ in the counterfactual world where the variables in $C^{B}$ are set equal to **c**^*B′*^. To achieve counterfactual fairness [whose definition we draw from the definitions in Kusner et al. (2017) and Zuo et al. (2022)], it is sufficient that, for all **c**^*A*^, **c**^*B*^, **c**^*B′*^, *x*,


$$\begin{array}{l} \mathbb{P}\left[{\hat{\phi }}_{C^{B}\leftarrow {\text{c}}^{B}}\left({\text{c}}^{A}\right)=x|{\text{c}}^{A},{\text{c}}^{B}\right]=\mathbb{P}\left[{\hat{\phi }}_{C^{B}\leftarrow {\text{c}}^{B\prime }}\left({\text{c}}^{A}\right)=x|{\text{c}}^{A},{\text{c}}^{B}\right] \end{array}$$

Now, under the assumptions in Section 2.1.4, the conditional independences in G imply that we have


$$\begin{array}{l} \mathbb{P}\left[\hat{\phi }\left({\text{c}}^{A}\right)=x|{\text{c}}^{A},{\text{c}}^{B}\right]=\mathbb{P}\left[\hat{\phi }\left({\text{c}}^{A}\right)=x\right],\forall {\text{c}}^{B} \end{array}$$

This gives us that


$$\begin{array}{l} \mathbb{P}\left[{\hat{\phi }}_{C^{B}\leftarrow {\text{c}}^{B\prime }}\left({\text{c}}^{A}\right)=x|{\text{c}}^{A},{\text{c}}^{B}\right]=\mathbb{P}\left[\hat{\phi }\left({\text{c}}^{A}\right)=x\right],\forall {\text{c}}^{A},{\text{c}}^{B},{\text{c}}^{B\prime },x \end{array}$$

which satisfies the counterfactual fairness condition.

### 5.2 Demographic parity

A common criterion for group-level fairness is Demographic Parity (Kusner et al., 2017). Demographic Parity (DP) requires that the distribution over actions remains the same irrespective of the value of the sensitive variables.

Demographic parity is a popular notion of fairness as it aligns well with most people's understanding of fairness and does not require strong assumptions such as the knowledge of the underlying causal graph to compute. As an example, if a loan decision policy has demographic parity with respect to gender, then the probability of granting a loan given a random male is the same as that for a random individual from any other gender group.

In our case, note that


$$\mathbb{P}\left[\hat{\phi }=x|{\text{c}}^{B}\right]=\underset{{\text{c}}^{A}}{\sum }\mathbb{P}\left[{\text{c}}^{A},\hat{\phi }\left(\text{c}\right)=x|{\text{c}}^{B}\right]$$

Formally, DP requires that


$$\mathbb{P}\left[\hat{\phi }=x|{\text{c}}^{B}\right]=\mathbb{P}\left[\hat{\phi }=x|{\text{c}}^{B\prime }\right],\;\forall {\text{c}}^{B\prime }$$

Our algorithm, however, does *not* guarantee DP even if $C^{B}⊇W$:


$$\begin{array}{l} \mathbb{P}\left[\hat{\phi }=x|{\text{c}}^{B}\right]=\underset{{\text{c}}^{A}}{\sum }\mathbb{P}\left[{\text{c}}^{A}|{\text{c}}^{B}\right]\cdot \mathbb{P}\left[\hat{\phi }\left({\text{c}}^{A}\right)=x\right] \end{array}$$

which may not equal ℙ[ϕ = *x*|**c**^*B′*^] since ℙ[**c**^*A*^|**c**^*B*^] may not equal ℙ[**c**^*A*^|**c**^*B′*^]. And since $C^{B}⊇W$, we cannot guarantee demographic parity.

However, we discuss one way through which DP can be achieved, but with a reduction in agent's performance. Specifically, we can achieve DP by ensuring that the agent acts according to a fixed policy irrespective of the value of **c**^*A*^. Intuitively, we construct a fixed policy that maximizes rewards given the agent's learned beliefs.

Specifically, let $\hat{\psi }\left(x,{\text{c}}^{A}\right)≜\hat{𝔼}\left[Y|do\left(x\right),{\text{c}}^{A}\right]$. Assume the fixed policy is probabilistic. Therefore, we're interested in a policy *q* which is a distribution over |val(*X*)|. Denoting *q*^(*x*)^ ≜ *q*(*x*), we solve the following optimization problem:


$$\begin{array}{l} <...,q^{\left(x\right)},...>=\\ \;argmax_{<...,q^{{\left(x\right)}^{\prime }},...>}\underset{x}{\sum }\left[q^{{\left(x\right)}^{\prime }}\left[\underset{{\text{c}}^{A}}{\sum }\hat{\mathbb{P}}\left[{\text{c}}^{A}\right]\cdot \hat{\psi }\left(x,{\text{c}}^{A}\right)\right]\right] \end{array}$$

subject to


$$\begin{array}{l} \underset{x}{\sum }q^{{\left(x\right)}^{\prime }}=1 \end{array}$$

It is easy to see that one global optimum to this involves assigning a probability of 1 to an action *x* that results in the largest value of $\underset{{\text{c}}^{A}}{\sum }\hat{\mathbb{P}}\left[{\text{c}}^{A}\right]\cdot \hat{\psi }\left(x,{\text{c}}^{A}\right)$. That is, choose *x* such that


$$\begin{array}{l} x=argmax_{x^{\prime }}\underset{{\text{c}}^{A}}{\sum }\hat{\mathbb{P}}\left[{\text{c}}^{A}\right]\cdot \hat{\psi }\left(x^{\prime },{\text{c}}^{A}\right) \end{array}$$

and let *q*(*x*) = 1, and *q*(*x*′) = 0, ∀*x*′ ≠ *x*. Note that this fixed policy would perform worser on expectation than the context-specific policy learned by the agent in the main part of the paper. This, however, is a cost that can be paid to achieve DP.

As an example of how this might be useful in practice, note that a recruitment agency might be more concerned (perhaps due to legal requirements) about ensuring that it does not discriminate against particular races, genders, etc., even if that leads to suboptimal allocations of jobs. The above approach ensures that such an entity can achieve demographic parity, even though it costs in terms of performance.

As a toy example, suppose there are just two contexts {0, 1} and two actions {0, 1} such that for context 0, the rewards for actions 0 and 1 are 10 and 0, respectively; and for context 1, the corresponding rewards are 0 and 9. Both contexts are equally likely. In this case, the above approach will choose action 0 always and achieve DP with respect to the context variable, but will receive 0 reward for context 1 which is far from optimal. We do not provide a full-fledged characterization of the performance-fairness tradeoffs and reserve that for future work.

## 6 Discussion and conclusion

Though exploitation of causal side information in multi-armed bandits has been relatively well-studied, its integration in contextual bandit settings remains much less investigated. This work presented only the second work in this area. Specifically, this paper proposed a new contextual bandit problem formalism where the agent, which has access to qualitative causal side information, can also actively obtain a table of experimental data in one shot, but at a cost and within a budget.

Further, most contextual bandit problems have been studied in the case when contexts are received from the environment. However, there are several real world settings such as marketing campaigns where targeted experiments are possible. This work is one of the very few works to study this setting.

We proposed a novel algorithm based on a new measure similar to entropy, and showed extensive empirical analysis of our algorithm's performance. We also showed theoretical results on soundness and regret. As demonstrated, smartly exploiting information leakage from the causal graph can yield significantly improved performance. Further, it is also possible to achieve certain notions of individual and group fairness, though it might come at a cost of reduced performance.

This work opens up various directions of future research. Fairness of machine learning models is one of the fast-growing areas of research due to the increased interest in responsible development of AI; it is worthwhile to design causal contextual bandit algorithms that meet population-level fairness criteria with minimal impact on performance. It is also useful to investigate more general approaches to fairness such as optimizing under general fairness constraints. Another useful direction is to allow the presence of unobserved confounders in the causal model; while the lack of unmeasured confounders is frequently assumed in causal bandits literature, removing that assumption can provide a qualitative improvement to the setting and make it more widely applicable. It is also interesting to investigate infinite action or context spaces (for example, domains that are continuous) as they often show up in real world scenarios. Another direction is to study contextual bandit algorithms that combine causal discovery with regret optimization can further expand the scope of application by entirely removing the need for a causal graph to be provided. Finally, it might be fruitful to investigate ways to combine one-shot algorithms with sequential algorithms to provide a more comprehensive approach, and to also look at evolving causal graphs.

## Data Availability

Publicly available datasets were analyzed in this study. This data can be found at: Zenodo, https://zenodo.org/, doi: 10.5281/zenodo.5540348.

## Footnotes

^1^Actions in a contextual bandit setting can also be interpreted as Pearl *do*() interventions (Zhang and Bareinboim, 2017; Lattimore et al., 2016); so we use the term “action” and “intervention” interchangeably.

^2^Similar results for multi-armed bandits for best arm identification problems are shown in works such as Lattimore et al. (2016).

^3^See: https://firebase.google.com/docs/ab-testing for an example.

^4^See: https://www.persado.com/articles/the-power-of-experimental-design-to-deliver-marketing-insights/ for an example.

^5^For example, see: https://support.google.com/google-ads/answer/1704368

^6^For example, see: https://scale.com/blog/netflix-recommendation-personalization

^7^By qualitative, we mean that the agent can know the causal *graph*, but not the conditional probability distributions of the variables. See Section 2.1 for a more detailed discussion.

^8^In contrast, in a standard contextual bandit setting, the agent would repeatedly observe **c**^*A*^, respond with an *x*, and observe outcomes.

^9^*do*(*X* = *x*) is a standard operation in causal inference that models interventions, where parents of *X* are removed, and *X* is set equal to *x*. See Pearl (2009b, 2019) for more discussion on the *do*() operation. *do*(*X* = *x*) is succinctly written *do*(*x*).

^10^That is, if $C^{A}$ contains all its ancestors.

^11^“Belief” is a commonly used term to mean the posterior distribution of the parameters of the distribution of interest (Russo et al., 2017).

^12^Information leakage arises from shared pathways in M; or equivalently, due to shared CPDs in the factorization of ℙ.

^13^ https://docs.scipy.org/doc/scipy/reference/generated/scipy.optimize.differential_evolution.html
