# Supplementary material for: Causal contextual bandits with one-shot data integration
Source: Front Artif Intell. 2024 Dec 6;7:1346700. doi: 10.3389/frai.2024.1346700 (PMC11659213; doi:10.3389/frai.2024.1346700)
Supplement: Supplementary file 1 [file Data_Sheet_1.pdf]

Supplementary Material

1 CODE

The code for the experiments is made available at `https://github.com/coba-paper/code`. The settings files in the code also contain the full parameterizations of all experiments. Please see `README.md` in the repository for details on how to run the experiments.

2 ADDITIONAL EXPERIMENTS AND DISCUSSION

This section provides some additional experiments and discussion that are not part of the main results, but provide more intuition.

2.1 Detailed plots related to Experiment 4

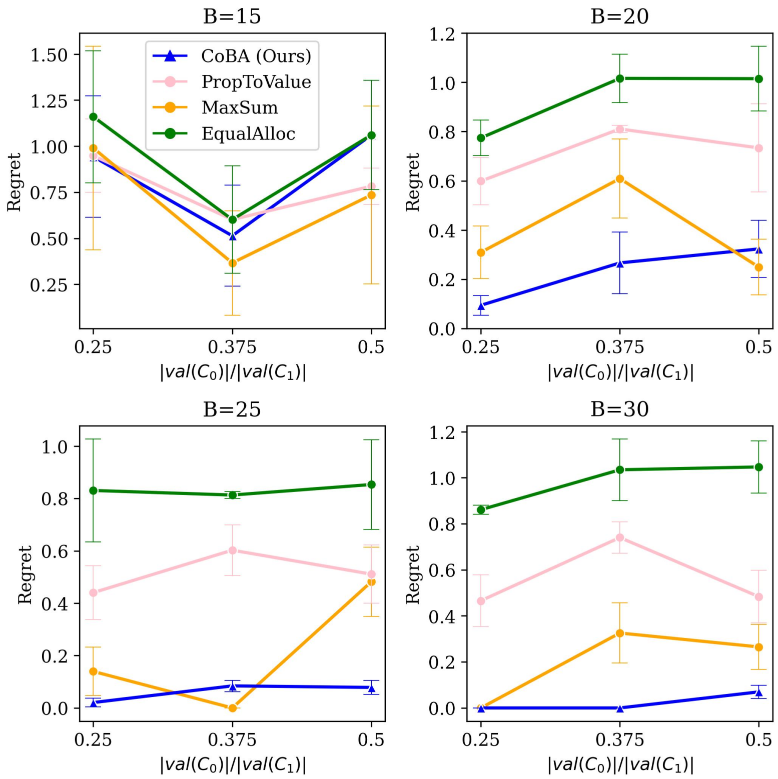

Figure S1. Detailed plots related to Experiment 4.

Figure S1 shows the breakdown of the results of Experiment 4 for each value of B. For $B = 15$, there is no clear pattern; this is because the budget is so small that they all tend to learn poor policies. But when we increase B, we see that our algorithm performs better when the graph is the most squeezed – like we had discussed in the main part of the paper. Further, in the most squeezed setting (lowest value of x-axis), our algorithm's difference from the next best algorithm is larger for smaller B, and decreases as B increases;

this is because a larger budget reduces the advantage that our algorithm gains from better utilization of information leakage.

2.2 Detailed plots related to Experiment 5

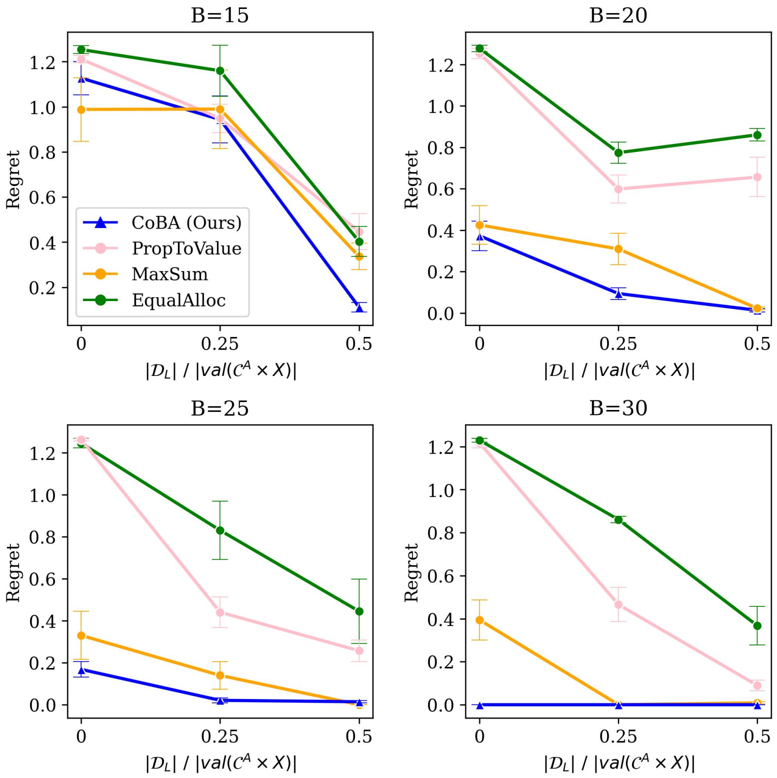

Figure S2. Detailed plots related to Experiment 5.

Figure S2 shows the breakdown of the results of Experiment 5 for each value of B. We see that for each value of B, as $\frac{\mathcal{D}_L}{\text{val}(\mathcal{C}^A \times X)}$ increases, regret decreases – similar to what happens in the aggregated plot. Further, as B increases, 0 regret is achieved faster – again in line with expectations.

2.3 Detailed plots related to Experiment 7

Figure S3 shows the breakdown of the results of Experiment 7a for each value of B. For $B = 15$, there is no clear pattern; this is because the budget is so small that they all tend to learn poor policies. But when we increase B, we see that all algorithms perform worser when $\mathcal{G}$ does not match the true underlying causal graph. Further, our algorithm continues retain its better performance over all baseline for all values of B.

2.4 Intuition for why EqualAlloc performs worse in Experiment 2

If we look at the results of Experiment 2, we see that that EqualAlloc performs worse only for low values of B, and significantly improves its performance once B becomes 35.

To understand the intuition, first note that Experiment 2 captures the 80-20 rule (while randomizing other aspects). It is easy to see that EqualAlloc overallocates to the lower-value 80% of contexts. PropToValue, on the other hand, allocates more to the 20%, and therefore performs better for lower budgets (i.e., makes better use of the small budget). However, as budget increases, PropToValue fails to explore well, causing

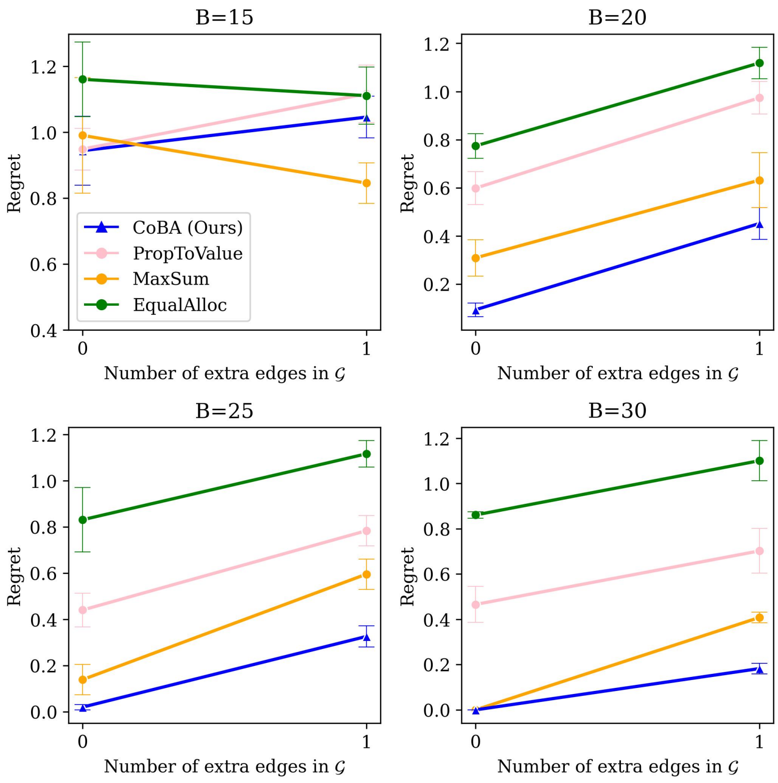

Figure S3. Detailed plots related to Experiment 7a.

EqualAlloc to outperform it. MaxSum outperforms EqualAlloc in lower budgets because allocations which allocate more to the higher-value contexts are also possible solutions for MaxSum, and therefore on average it performs better than EqualAlloc which overallocates to the low-value contexts consistently. However, as B becomes larger, we can see that EqualAlloc catches up.
